# Standardized multi-omics of Earth’s microbiomes reveals microbial and metabolite diversity

**DOI:** 10.1038/s41564-022-01266-x

**Published:** 2022-11-28

**Authors:** Justin P. Shaffer, Louis-Félix Nothias, Luke R. Thompson, Jon G. Sanders, Rodolfo A. Salido, Sneha P. Couvillion, Asker D. Brejnrod, Franck Lejzerowicz, Niina Haiminen, Shi Huang, Holly L. Lutz, Qiyun Zhu, Cameron Martino, James T. Morton, Smruthi Karthikeyan, Mélissa Nothias-Esposito, Kai Dührkop, Sebastian Böcker, Hyun Woo Kim, Alexander A. Aksenov, Wout Bittremieux, Jeremiah J. Minich, Clarisse Marotz, MacKenzie M. Bryant, Karenina Sanders, Tara Schwartz, Greg Humphrey, Yoshiki Vásquez-Baeza, Anupriya Tripathi, Laxmi Parida, Anna Paola Carrieri, Kristen L. Beck, Promi Das, Antonio González, Daniel McDonald, Joshua Ladau, Søren M. Karst, Mads Albertsen, Gail Ackermann, Jeff DeReus, Torsten Thomas, Daniel Petras, Ashley Shade, James Stegen, Se Jin Song, Thomas O. Metz, Austin D. Swafford, Pieter C. Dorrestein, Janet K. Jansson, Jack A. Gilbert, Rob Knight, the Earth Microbiome Project 500 (EMP500) Consortium, Lars T. Angenant, Alison M. Berry, Leonora S. Bittleston, Jennifer L. Bowen, Max Chavarría, Don A. Cowan, Dan Distel, Peter R. Girguis, Jaime Huerta-Cepas, Paul R. Jensen, Lingjing Jiang, Gary M. King, Anton Lavrinienko, Aurora MacRae-Crerar, Thulani P. Makhalanyane, Tapio Mappes, Ezequiel M. Marzinelli, Gregory Mayer, Katherine D. McMahon, Jessica L. Metcalf, Sou Miyake, Timothy A. Mousseau, Catalina Murillo-Cruz, David Myrold, Brian Palenik, Adrián A. Pinto-Tomás, Dorota L. Porazinska, Jean-Baptiste Ramond, Forest Rowher, Taniya RoyChowdhury, Stuart A. Sandin, Steven K. Schmidt, Henning Seedorf, Ashley Shade, J. Reuben Shipway, Jennifer E. Smith, James Stegen, Frank J. Stewart, Karen Tait, Torsten Thomas, Yael Tucker, Jana M. U’Ren, Phillip C. Watts, Nicole S. Webster, Jesse R. Zaneveld, Shan Zhang

**Affiliations:** 1 Department of Pediatrics, School of Medicine, University of California San Diego, La Jolla, CA USA; 2 Collaborative Mass Spectrometry Innovation Center, University of California San Diego, La Jolla, CA USA; 3 Skaggs School of Pharmacy and Pharmaceutical Sciences, University of California San Diego, La Jolla, CA USA; 4 Northern Gulf Institute, Mississippi State University, Starkville, MS USA; 5 Ocean Chemistry and Ecosystems Division, Atlantic Oceanographic and Meteorological Laboratory, National Oceanic and Atmospheric Administration, Miami, FL USA; 6 Department of Ecology and Evolutionary Biology, Cornell University, Ithaca, NY USA; 7 Department of Bioengineering, University of California San Diego, La Jolla, CA USA; 8 Earth and Biological Sciences Directorate, Pacific Northwest National Laboratory, Richland, WA USA; 9 Center for Microbiome Innovation, Jacobs School of Engineering, University of California San Diego, La Jolla, CA USA; 10 IBM Research, T.J. Watson Research Center, Yorktown Heights, NY USA; 11 Scripps Institution of Oceanography, University of California San Diego, La Jolla, CA USA; 12 School of Life Sciences, Arizona State University, Tempe, AZ USA; 13 Biodesign Center for Fundamental and Applied Microbiomics, Arizona State University, Tempe, AZ USA; 14 Bioinformatics and Systems Biology Program, Jacobs School of Engineering, University of California San Diego, La Jolla, CA USA; 15 Center for Computational Biology, Flatiron Institute, Simons Foundation, New York, NY USA; 16 Chair for Bioinformatics, Friedrich Schiller University, Jena, Germany; 17 College of Pharmacy and Integrated Research Institute for Drug Development, Dongguk University, Gyeonggi-do, Korea; 18 Department of Chemistry, University of Connecticut, Storrs, CT USA; 19 Department of Computer Science, University of Antwerp, Antwerp, Belgium; 20 IBM Research Europe, Daresbury, UK; 21 IBM Research, Almaden Research Center, San Jose, CA USA; 22 Environmental Genomics and Systems Biology, Lawrence Berkeley National Laboratory, Berkeley, CA USA; 23 Department of Virus and Microbiological Special Diagnostics, Statens Serum Institute, Copenhagen, Denmark; 24 Department of Chemistry and Bioscience, Aalborg University, Aalborg, Denmark; 25 Centre for Marine Science and Innovation, School of Biological, Earth and Environmental Science, The University of New South Wales, Sydney, New South Wales Australia; 26 Interfaculty Institute of Microbiology and Infection Medicine, University of Tübingen, Tübingen, Baden-Württemberg Germany; 27 Department of Microbiology and Molecular Genetics, Michigan State University, East Lansing, MI USA; 28 Department of Computer Science and Engineering, Jacobs School of Engineering, University of California San Diego, La Jolla, CA USA; 29 University of Tübingen, Tübingen, Baden-Württemberg Germany; 30 University of California, Davis, Davis, CA USA; 31 Boise State University, Boise, ID USA; 32 Northeastern University, Boston, MA USA; 33 University of Costa Rica, San José, Costa Rica; 34 CENIBiot, San José, Costa Rica; 35 University of Pretoria, Pretoria, South Africa; 36 Harvard University, Cambridge, MA USA; 37 Universidad Politécnica de Madrid, Instituto Nacional de Investigación y Tecnología Agraria y Alimentaria, Madrid, Spain; 38 University of California San Diego, La Jolla, CA USA; 39 Janssen Research and Development, San Diego, CA USA; 40 Louisiana State University, Baton Rouge, LA USA; 41 University of Jyväskylä, Jyväskylä, Finland; 42 University of Pennsylvania, Philadelphia, PA USA; 43 The University of Sydney, Sydney, New South Wales Australia; 44 Texas Technology University, Lubbock, TX USA; 45 University of Wisconsin, Madison, WI USA; 46 Colorado State University, Fort Collins, CO USA; 47 Temasek Life Sciences Laboratory, Singapore, Singapore; 48 Oregon State University, Corvallis, OR USA; 49 University of Florida, Gainesville, FL USA; 50 Pontificia Universidad Católica de Chile, Santiago, Chile; 51 San Diego State University, San Diego, CA USA; 52 Pacific Northwest National Laboratory, Richland, WA USA; 53 University of Maryland, College Park, MD USA; 54 Department of Ecology and Evolutionary Biology, University of Colorado at Boulder, Boulder, CO USA; 55 National University of Singapore, Singapore, Singapore; 56 University East Lansing, East Lansing, MI USA; 57 University of Plymouth, Plymouth, UK; 58 University of Massachusetts Amherst, Amherst, MA USA; 59 Montana State University, Bozeman, MT USA; 60 Plymouth Marine Laboratory, Plymouth, UK; 61 University of New South Wales, Sydney, New South Wales Australia; 62 National Energy Technology Laboratory, Pittsburgh, PA USA; 63 University of Arizona, Tucson, AZ USA; 64 Australian Institute of Marine Science, Townsville, Queensland Australia; 65 University of Queensland, Brisbane, Queensland Australia; 66 University of Washington Bothell, Bothell, WA USA

**Keywords:** Microbial ecology, Chemical ecology, Metabolomics, Next-generation sequencing, Cheminformatics

## Abstract

Despite advances in sequencing, lack of standardization makes comparisons across studies challenging and hampers insights into the structure and function of microbial communities across multiple habitats on a planetary scale. Here we present a multi-omics analysis of a diverse set of 880 microbial community samples collected for the Earth Microbiome Project. We include amplicon (16S, 18S, ITS) and shotgun metagenomic sequence data, and untargeted metabolomics data (liquid chromatography-tandem mass spectrometry and gas chromatography mass spectrometry). We used standardized protocols and analytical methods to characterize microbial communities, focusing on relationships and co-occurrences of microbially related metabolites and microbial taxa across environments, thus allowing us to explore diversity at extraordinary scale. In addition to a reference database for metagenomic and metabolomic data, we provide a framework for incorporating additional studies, enabling the expansion of existing knowledge in the form of an evolving community resource. We demonstrate the utility of this database by testing the hypothesis that every microbe and metabolite is everywhere but the environment selects. Our results show that metabolite diversity exhibits turnover and nestedness related to both microbial communities and the environment, whereas the relative abundances of microbially related metabolites vary and co-occur with specific microbial consortia in a habitat-specific manner. We additionally show the power of certain chemistry, in particular terpenoids, in distinguishing Earth’s environments (for example, terrestrial plant surfaces and soils, freshwater and marine animal stool), as well as that of certain microbes including *Conexibacter woesei* (terrestrial soils), *Haloquadratum walsbyi* (marine deposits) and *Pantoea dispersa* (terrestrial plant detritus). This Resource provides insight into the taxa and metabolites within microbial communities from diverse habitats across Earth, informing both microbial and chemical ecology, and provides a foundation and methods for multi-omics microbiome studies of hosts and the environment.

## Abstract (web summary)

This Resource combines amplicon sequencing, shotgun metagenomic sequencing and untargeted metabolomics to provide a global view of microbial–metabolite associations across Earth’s environments.

## Main

A major goal in microbial ecology is to understand structure in microbial communities, how this is related to microbial taxonomic, phylogenetic and functional composition, and how those relationships vary across space and time. As any single study is not able to sample all environments repeatedly to allow for such inferences, fostering the use of standardized methods that permit meta-analysis across distinct studies is of utmost importance^1–4^. Initial efforts focused on standardized protocols for 16S ribosomal RNA (rRNA) sequencing of bacterial/archaeal communities provided insight into how communities structure in the environment, supporting strong axes of separation of microbes along gradients of host association and salinity^1,5^. More recent efforts focused on shotgun metagenomics data^6–9^ have begun to provide additional insight regarding functional potential across environments^10–14^, and the current state-of-the-art methods employ multi-omics approaches including metagenomics, transcriptomics, proteomics and/or metabolomics^15–24^.

Microbes produce diverse secondary metabolites that perform vital functions from communication to defence^25–27^ and can benefit human health and environmental sustainability^28–34^. Whereas metagenome mining and transcriptomics are powerful ways to characterize function in microbial communities^10,14,24^, a more powerful approach to understanding functional diversity is to generate chemical evidence that confirms the presence of metabolites^19–21^ and accurately describes their distribution across Earth. Here we present an approach that directly assesses the presence and relative abundance of metabolites, and provides an accurate description of metabolite profiles in microbial communities across Earth’s environments. Although several studies have previously employed tandem metagenomics and metabolomics^22,23,35–40^, many employed relatively limited technical methods or profiled a relatively small number of classes of metabolites^23,35,40^, preventing comparison across studies that could expand our understanding. Further, several previous studies are limited in scope to a single environment or habitat^20,23,24,35–39^. Our work goes substantially beyond what has been reported previously regarding multi-omics analysis of microbial communities using metagenomics and metabolomics, by including multiple ecosystems. The approach we apply complements metagenomics with a direct survey of secondary metabolites using untargeted metabolomics.

Liquid chromatography with untargeted tandem mass spectrometry (LC–MS/MS) is a versatile method that detects tens of thousands of metabolites in biological samples^19^. Although LC–MS/MS metabolomics has historically suffered from low metabolite annotation rates when applied to non-model organisms, recent computational advances can systematically assign chemical classes to metabolites using their fragmentation spectra^41^. Untargeted mass-spectrometry-based metabolomics provides the relative abundance (that is, intensity) of each metabolite detected across samples rather than just counts of unique structures (that is, presence/absence data), and thus provides a direct readout of the surveyed environment, complementing a purely genomics-based approach. Although there is a clear need to use untargeted metabolomics to quantify the metabolic activities of microbiota, the approach has been limited by the challenge of distinguishing the secondary metabolites produced exclusively by microbes from other compounds detected in the environment (for example, those produced by multicellular hosts). To resolve this bottleneck, we devised a computational method for recognizing and annotating putative secondary metabolites of microbial origin from fragmentation spectra (see Online Methods).

We used this methodology to quantify microbial secondary metabolites from diverse microbial communities from the Earth Microbiome Project (EMP, http://earthmicrobiome.org). The EMP was founded in 2010 to sample Earth’s microbial communities at unprecedented scale, in part to advance our understanding of biogeographic processes that shape community structure. To avoid confusion with terminology, we define ‘microbial community’ as consisting of members of the domains Bacteria and Archaea. To build on the first analysis of the EMP archive focused on profiling bacterial and archaeal 16S rRNA^1^, we crowd-sourced a previously undescribed set of roughly 900 samples from the scientific community specifically for multi-omics analysis. We expanded the scalable framework of the EMP to include standardized methods for shotgun metagenomic sequencing and untargeted metabolomics for cataloguing microbiota globally. As a result, we provide a rich resource for addressing outstanding questions and to serve as a benchmark for acquiring additional data. To provide an example for using this resource, we present a multi-omics analysis of this undescribed sample set, tracking not just individual sequences but also genomes and metabolites. Our analysis includes diverse studies with sample types classified using an updated and standardized environmental ontology, describes large-scale ecological patterns and explores important questions in microbial ecology.

Specifically, we explore the hypothesis that ‘everything is everywhere but the environment selects’^42–46^. We predict that although most major classes of metabolites have cosmopolitan distributions^14^, their relative abundances will vary strongly among different environments. Therefore, whereas the presence/absence of metabolites alone may show profiles that are relatively uniform across samples, their relative abundances will provide great power in distinguishing among habitats. We predict that similar to microbes^1^, metabolites will exhibit both turnover and nestedness across habitats. Furthermore, we expect variation in metabolite profiles among environments to be in part driven by variation in microbial community composition. Therefore, we explore the hypothesis that metabolite alpha- and beta-diversity will be strongly correlated with microbial diversity. We anticipate strong positive relationships between microbial diversity and metabolite diversity, but that environmental similarity based on microbial composition may be distinct from that based on metabolite composition. We suspect that this is in part due to deterministic processes unique to microbial community assembly and similarity in metabolite profiles across the microbial phylogeny^47–49^. Regardless, if profiles for metabolites and microbes are habitat-specific, we predict that certain members can be used to classify samples among environments. We also predict that metabolites will co-occur with specific microbial taxa such that metabolite–microbe pairs can be described as features in the environment that define specific habitats.

## Results

### A resource for multi-omics in microbial ecological research

**Fig. 1:**
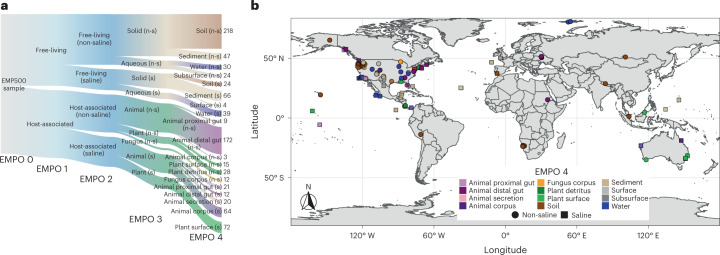
Environment type and provenance of samples. **a**, Distribution of samples (*n* = 880) among the Earth Microbiome Project Ontology (EMPO version 2) categories. EMPO recognizes strong axes of variation in microbial communities, and thus organizes all microbial environments (level 4) on the basis of host association (level 1), salinity (level 2), host taxon (for host-associated) or phase (free-living) (level 3). For EMPO 3 and EMPO 4: n-s, non-saline; s, saline. Colours indicate environments. Numbers indicate sample counts for each environment. Made with JSFiddle. **b**, Geographic distribution of samples with points coloured by EMPO 4. Points are transparent to highlight cases where multiple samples derive from a single location. We note here that our intent was to sample across environments rather than geography, in part because we previously showed that microbial community composition is more influenced by the former rather than the latter, but also to motivate finer-grained geographic exploration as sample analyses decrease in cost. Extensive information about each sample set is described in Supplementary Table 1. Made with Natural Earth.

Here we generated data for 880 environmental samples that span 19 major environments contributed by 34 principal investigators as part of the Earth Microbiome Project 500 (EMP500). The EMP500 is a previously unreported sample set for multi-omics protocol development and data exploration (Fig. 1 and Supplementary Table 1). To normalize sample collection for this and future studies, we updated and followed the existing Earth Microbiome Project (EMP) sample submission guide (https://earthmicrobiome.org/protocols-and-standards/emp500-sample-submission-guide/)^50^, which we highlight here to encourage its use. In parallel, we followed standardized protocols for sample collection, sample tracking, sample metadata curation, sample shipping and data release, which are also detailed on the EMP website (https://earthmicrobiome.org/protocols-and-standards/) and described here (see Online Methods). Importantly, we updated the previous EMP Metadata Guide to accommodate the EMP500 sampling design as well as updates to other standardized ontologies (see Online Methods), including the Earth Microbiome Project Ontology (EMPO). EMPO classifies microbial environments (level 4) on the basis of host association (level 1), salinity (level 2), host kingdom (if host-associated) or phase (if free-living) (level 3) (Fig. 1a). EMPO now recognizes an important split within host-associated samples representing saline and non-saline environments (Fig. 1a) not detected in the EMP’s previous analysis of 16S rRNA from a separate set of <23,000 samples^1^.

**Extended Data Fig. 1:**
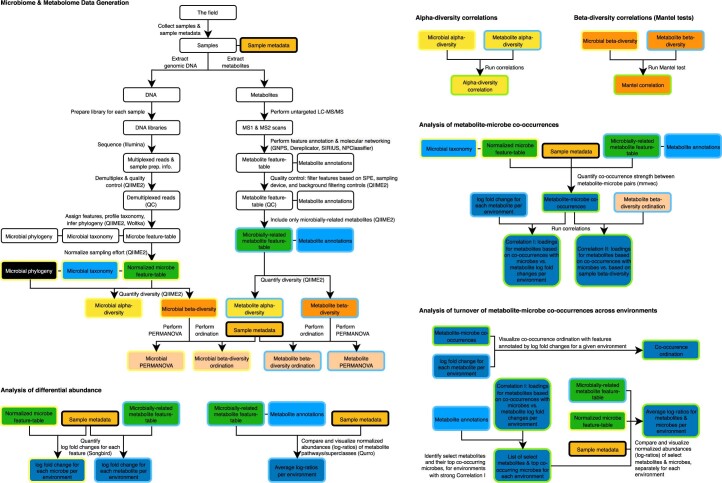
Diagrammatic overview of multi-omics analyses performed using the EMP500 dataset. The process begins with data generation for both the microbiome and metabolome, which is then followed by analysis of differential abundance of both microbial taxa and microbially-related metabolites across environments. To begin multi-omics integration, correlations between alpha- and beta-diversity are explored, followed by explicit co-occurrence analysis of metabolite-microbe pairs. The results from analysis of co-occurrence are then combined with those from analysis of differential abundance, to reveal strong patterns of metabolite-microbe turnover across environments. Throughout the diagram, artifacts derived from microbial data are outlined in yellow, those derived from metabolite data are outlined in blue, and those derived from co-occurrence analysis are outlined in green.

For the majority of samples, we successfully generated data for bacterial and archaeal 16S rRNA, eukaryotic 18S rRNA, internal transcribed spacer (ITS) 1 of the fungal ITS region, bacterial full-length rRNA operon, shotgun metagenomics and untargeted metabolomics (that is, LC–MS/MS and gas chromatography coupled with mass spectrometry (GC–MS)) (Supplementary Table 2). To foster exploration of this previously unreported dataset, we have made the raw sequence and metabolomics data publicly available through Qiita (https://qiita.ucsd.edu; study ID: 13114)^51^ and GNPS (https://gnps.ucsd.edu; MassIVE IDs: MSV000083475, MSV000083743)^52^, respectively. We also provide complete protocols for laboratory and computational workflows for both metagenomics and metabolomics data for use by the broader community (available on GitHub at https://github.com/biocore/emp/blob/master/methods/methods_release2.md). We hope that the dataset and workflows presented here serve as useful tools for others, in addition to providing a framework for launching additional future studies. As an example of the utility of the dataset for addressing important questions in microbial community ecology, we present an analysis of microbially related metabolites and microbe–metabolite co-occurrences across Earth’s environments (Extended Data Fig. 1).

### Metabolite intensities reveal habitat-specific distributions

**Fig. 2:**
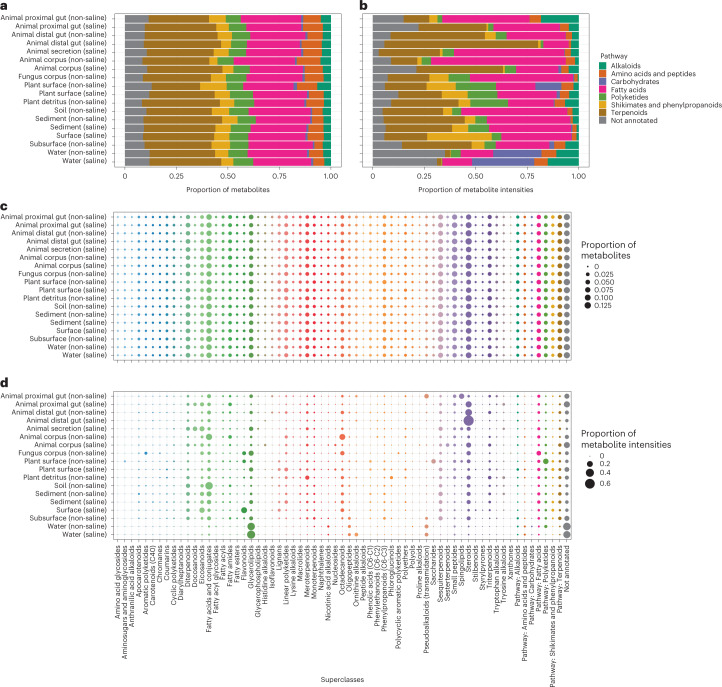
Distribution of microbially related secondary metabolite pathways and superclasses among environments. **a**–**d**, Individual metabolites are represented by their higher-level classifications. Both chemical pathway and chemical superclass annotations are shown on the basis of presence/absence (**a**,**c**) and relative intensities (**b**,**d**) of molecular features, respectively. For superclass annotations in **c** and **d**, we included pathway annotations (when possible) for metabolites where superclass annotations were not available, and colours identify superclasses and pathways.

**Extended Data Fig. 2:**
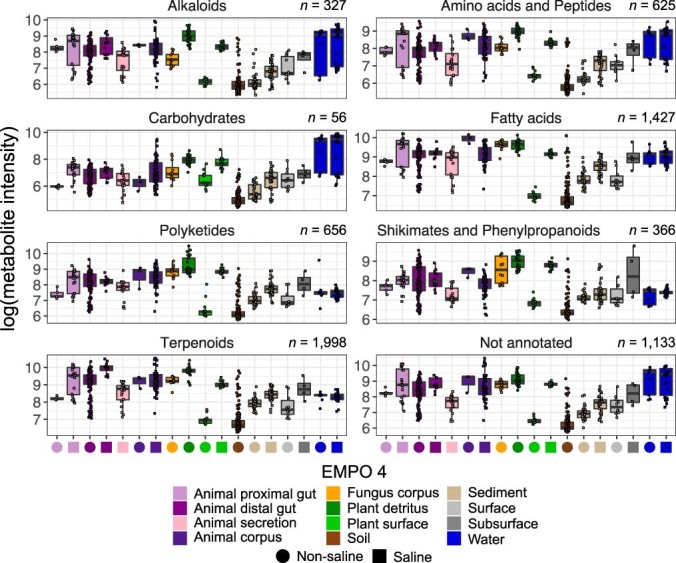
Relative abundance of microbially-related metabolite pathways, highlighting among-sample variation for each environment. These data are shown as a complement to those in Fig. 2b of the main text. We note that as abundance data were not normalized (for example, by using log-ratios as in Fig. 3a), caution should be used in interpreting differences among environments. Boxplots are in the style of Tukey, where the center line indicates the median, lower and upper hinges the first- and third quartiles, respectively, and each whisker 1.5 x the interquartile range (IQR) from its respective hinge. For each panel, *n* = 618 biologically independent samples, and the number of metabolites per pathway is shown.

In total, we generated untargeted metabolomics data (that is, LC–MS/MS) for 618 of 880 samples (Supplementary Table 2), resulting in 52,496 unique molecular structures, or metabolites, across all samples. We then refined that dataset to include only putative, microbially related metabolites (that is, defined as being produced, modified by, or otherwise associated with a microbe), resulting in 6,588 metabolites across all samples (12.55% of all metabolites). Focusing on this subset, we found that although the presence/absence of major classes of microbially related metabolites is relatively conserved across habitats, their relative intensities (that is, analogous to relative abundances for microbes) reveal specific chemistry that is lacking or enriched in particular environments (Fig. 2 and Extended Data Fig. 2).

**Fig. 3:**
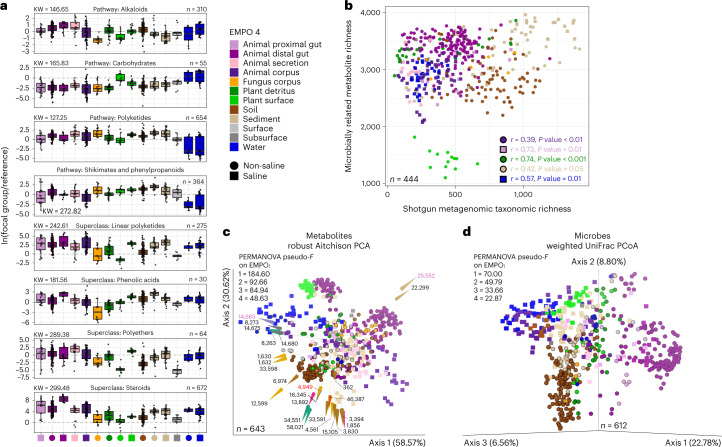
Structural-level associations between microbially related secondary metabolites and specific environments. **a**, Differential abundance of metabolites across environments. For each panel, the *y* axis represents the natural log-ratio of the intensities of ingroup metabolites divided by the intensities of reference group metabolites (that is, pathway reference: Amino acids and peptides, *n* = 615; superclass reference: Flavonoids, *n* = 42). The number of metabolites in each ingroup and the chi-squared statistic from a Kruskal–Wallis (KW) test for differences across environments are shown. For each test, *n* = 606 samples and *P* < 2.2 × 10^−16^. Boxplots are Tukey’s, where the centre indicates the median, lower and upper hinges the first and third quartiles, respectively, and each whisker is 1.5× the interquartile range (IQR) from its hinge. **b**, Relationship between metabolite richness and microbial taxon richness, with significant correlations noted. *P* values are from two-tailed tests and were adjusted using the Benjamini-Hochberg procedure. **c**, Turnover in composition of metabolites across environments, visualized using RPCA, showing samples separated on the basis of metabolite abundances. Shapes represent samples. Arrows represent metabolites and are coloured by chemical pathway. The direction and magnitude of each arrow corresponds to the correlation between the metabolite’s abundance and the ordination axes. Samples close to arrow heads have strong positive associations, samples at arrow origins have no association, and those beyond arrow origins have strong negative associations. Metabolites are described in Supplementary Table 4. Metabolites annotated in red and purple were also highly differentially abundant across environments (Supplementary Table 3), and those in purple were also identified as important in co-occurrence analyses (Fig. 4). **d**, Turnover in composition of microbial taxa across environments, visualized using PCoA of weighted UniFrac distances. For **c** and **d**, results from PERMANOVA (999 permutations) for each level of EMPO are shown (all tests had *P* = 0.001; group sizes for metabolites: *k*_EMPO1_ = 2, *k*_EMPO2_ = 4, *k*_EMPO3_ = 9, *k*_EMPO4_ = 18; group sizes for microbial taxa: *k*_EMPO1_ = 2, *k*_EMPO2_ = 4, *k*_EMPO3_ = 9, *k*_EMPO4_ = 19). Sample sizes in **a** refer to metabolites, but in all other panels refer to samples.

Importantly, when considering differences in the relative intensities of all microbially related metabolites, profiles for each habitat were so distinct that we could identify particular metabolites whose abundances were significantly enriched in certain environments (Fig. 3a and Supplementary Table 3). For example, metabolites annotated as carbohydrates (that is, excluding glycosides) were enriched in aquatic samples (log fold change (LFC)_Water (non-saline)_ = 0.31 ± 1.22, LFC_Water (saline)_ = 0.54 ± 1.45) (Fig. 3a). Similarly, sediment, marine plant surface and fungal samples were enriched in polyketides (LFC_Sediment (non-saline)_ = 1.69 ± 0.64, LFC_Sediment (saline)_ = 1.56 ± 1.11, LFC_Plant surface (saline)_ = 1.22 ± 0.35, LFC_Fungus corpus (non-saline)_ = 1.68 ± 1.10) and soil, lake sediment and marine plant surface samples were enriched in shikimates and phenylpropanoids (LFC_Sediment (non-saline)_ = 1.90 ± 0.69, LFC_Soil (non-saline)_ = 1.33 ± 0.65, LFC_Plant surface (saline)_ = 1.09 ±0.43) (Fig. 3a).

The total number of distinct metabolites (that is, richness) also varied strongly across environments (Fig. 3b). We note that whereas saline sediments were most rich, the surfaces of terrestrial plants were especially lacking in metabolite diversity (Fig. 3b). This contrasted with metabolite diversity in detritus of terrestrial plants, which was also high (Fig. 3b).

When considering the identity and relative intensity of each metabolite in the analysis of beta-diversity, we observed a separation of samples based on host association and salinity (permutational multivariate analysis of variance (PERMANOVA) for EMPO 2: pseudo-*F* = 92.66, *P* = 0.001), and among specific environments (PERMANOVA for EMPO 4: pseudo-*F* = 48.63, *P* = 0.001). We also observed specific environments clustering in ordination space and identified certain metabolite features that differentiate all samples (Fig. 3c and Supplementary Table 4). For the latter, we identified three metabolites also listed among the 10 most differentially abundant metabolites for each environment (Supplementary Table 3): one chalcone associated with the surfaces of terrestrial plants (C_13_H_10_O, ID: 4949), one glycerolipid associated with freshwater (C_28_H_58_O_15_, ID: 14665) and one cholane steroid associated with the distal guts of terrestrial animals (C_24_H_34_O_2_, ID: 25552) (Fig. 3c). As the separation of samples based on metabolite profiles appeared to mirror those based on microbial taxa (Fig. 3c,d), we additionally explored our shotgun metagenomics data.

### Correlation between metabolite and microbial alpha-diversity

We first explored whether metabolite alpha-diversity was related to microbe alpha-diversity. We found significant positive correlations between metabolite richness and microbial taxon richness across all samples (*r* = 0.20, *P* < 0.001), within host-associated samples (*r* = 0.19, *P* < 0.01), within free-living samples (*r* = 0.18, *P* < 0.05) and for certain environments: Animal proximal gut (saline) (*r* = 0.73, *P* < 0.01), Plant detritus (non-saline) (*r* = 0.74, *P* < .001), Sediment (non-saline) (*r* = 0.42, *P* = 0.05) and Water (saline) (*r* = 0.57, *P* = 0.01) (Fig. 3b and Supplementary Table 6). We observed non-significant trends in correlations for Plant surface (non-saline) (*r* = −0.36, *P* = 0.2) and Sediment (saline) (*r* = 0.27, *P* = 0.1) (Fig. 3b and Supplementary Table 6). Relationships for other environments were weaker (Fig. 3b and Supplementary Table 6). Sediment samples had the highest alpha-diversity of both microbial taxa and metabolites (Fig. 3b). Correlations with metabolite richness were weaker when using Faith’s phylogenetic diversity (PD) and weighted Faith’s PD for microbial taxa (Supplementary Table 6).

### Turnover and nestedness are related to the environment

**Extended Data Fig. 3:**
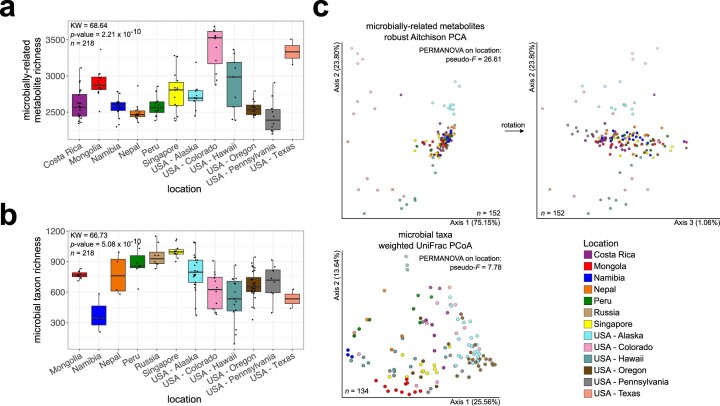
Microbially-related metabolite and microbial taxon composition among geographic locations for all non-saline soil samples. **a**, Metabolite richness. **b**, Microbe richness. For **a** and **b**, the chi-squared statistic from a Kruskal-Wallis rank sum test for differences in richness across environments is shown (that is, each test had *p*-value < 2.2 x 10^-16^). **c**, Beta-diversity based on metabolites (upper panel) and microbes (lower panel). Results from PERMANOVA tests (*n* = 999 permutations) for variance explained by salinity as well as each level of EMPO are shown; *p*-value = 0.001 for all tests.

**Extended Data Fig. 4:**
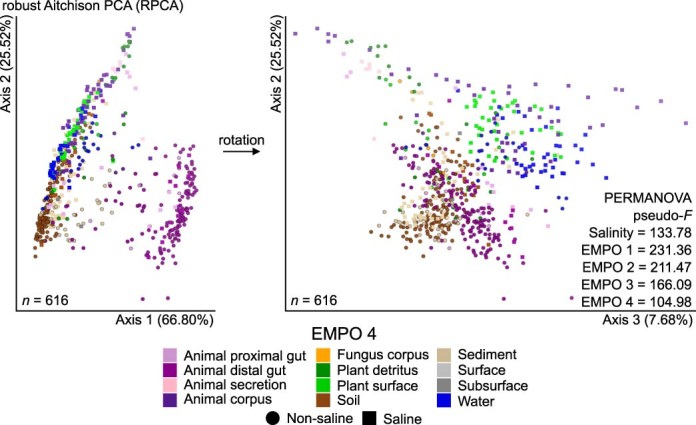
Clustering of samples by environments highlighting beta-diversity based on shotgun metagenomics data for microbial functions. Robust Aitchison PCA with samples colored by EMPO 4 and shaped by salinity. Features are KEGG ECs (that is, enzymes). Results from PERMANOVA tests (*n* = 999 permutations) for variance explained by salinity as well as each level of EMPO are shown; *p*-value = 0.001 for all tests.

Next, we examined whether metabolite diversity among environments (that is, beta-diversity) was driven by either turnover (that is, the replacement of features) or nestedness (gain/loss of features leading to differences in richness)^1,53^. We first looked at turnover. We already noted similarity in the clustering of samples by environment between microbially related metabolite and microbial taxon datasets (Fig. 3c,d). We also observed a strong correlation between sample–sample distances based on metabolites vs microbial taxa (Table 1). Interestingly, we observed a stronger effect of salinity when comparing samples on the basis of microbial taxa vs metabolites (PERMANOVA on salinity: pseudo-*F* = 40.94 for microbes vs 8.25 for metabolites, *P* = 0.001 for both tests) (Fig. 3c,d). Furthermore, when focusing on the separation of samples within a single environment such as soil, we observed much more variability between metabolite and microbial taxon datasets (Mantel *r* = 0.32 for soil vs 0.43 for all environments, *P* = 0.001 for both tests). This highlights the unique composition among soil samples from distinct locations (Extended Data Fig. 3), and also the insight that was gained from analysis at different scales (that is, only soils vs all habitats). To assess whether metabolite profiles were more similar to those for microbial taxa vs microbial functions, we annotated our metagenomic reads to profile enzymes. We found the separation of samples based on microbial functions to be unique and largely driven by animal gut samples as compared to separation based on either metabolites or microbial taxa (Extended Data Fig. 4). However, correlations in sample–sample distances between microbial functional data and other datasets were strong (Table 1).

**Table 1:** Mantel test results comparing data layers generated for the EMP500 samples

Dataset 1	Dataset 2	*n*	Spearman rho	*P* value
LC–MS/MS	GC–MS	401	0.13	0.001
	Metagenomics (taxa)	454	***0.43***	0.001
	Metagenomics (function)	440	**0.32**	0.001
	16S	477	**0.27**	0.001
	18S	340	0.07	0.2
	ITS	373	0.07	0.006
	full-length rRNA operon	181	**0.34**	0.001
GC–MS	Metagenomics (taxa)	331	0.07	0.002
	Metagenomics (function)	327	0.11	0.001
	16S	349	**0.22**	0.001
	18S	280	0.08	0.004
	ITS	269	0.09	0.001
	full-length rRNA operon	168	0.11	0.001
Metagenomics (taxa)	Metagenomics (function)	564	***0.53***	0.001
	16S	538	***0.51***	0.001
	18S	363	−0.002	0.9
	ITS	423	0.16	0.001
	full-length rRNA operon	235	***0.48***	0.001
Metagenomics (function)	16S	538	***0.58***	0.001
	18S	375	−0.02	0.4
	ITS	413	**0.22**	0.001
	full-length rRNA operon	239	***0.55***	0.001
16S	18S	414	0.09	0.001
	ITS	463	0.09	0.001
	full-length rRNA operon	215	***0.51***	0.001
18S	ITS	385	−0.05	0.1
	full-length rRNA operon	173	0.006	0.8
ITS	full-length rRNA operon	171	0.02	0.6

Note the strong relationships between the metabolomics data (that is, LC–MS/MS and GC–MS) and the sequence data from Bacteria and Archaea (that is, shotgun metagenomics, 16S and full-length rRNA operon) as compared to relationships between metabolomics data and sequence data from eukaryotes (that is, 18S and ITS). There are also strong relationships between difference sequence data from Bacteria and Archaea (rho > 0.2 in bolded font; >0.4 in bolded italics; >0.5 additionally underlined).

**Extended Data Fig. 5:**
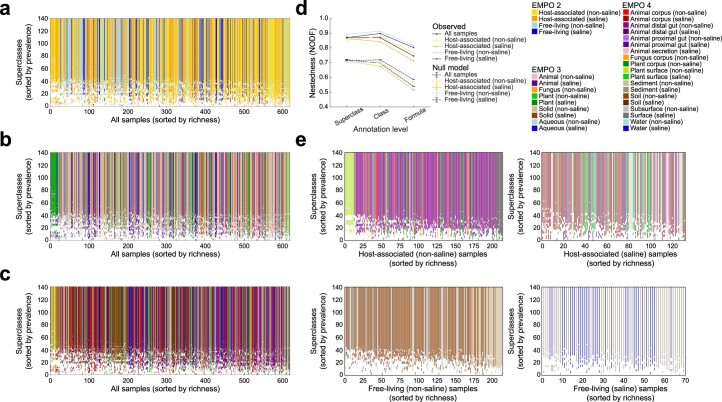
Nestedness of community composition based on microbially-related metabolites. **a**, Presence-absence of superclasses across samples, with superclasses (rows) sorted by prevalence and samples (columns, *n =* 618) sorted by richness. With increasing sample richness, superclasses tended to be gained but not lost (SES = 108.61, *p*-value < 0.0001 vs. a null model from a two-tailed test; nestedness measure based on overlap and decreasing fills [NODF] statistic = 0.87). Samples are colored by EMPO 2. **b**, As in **a** but with samples colored by EMPO 3. **c**, As in **a** but with samples colored by EMPO 4. **d**, Nestedness as a function of annotation level, from superclass to molecular formula, across all samples and within environments based on EMPO 2. Also shown are median null model NODF scores (± s.d.) for all samples, as well as samples at each level of EMPO 2. NODF measures the average fraction of metabolites from less diverse communities that occur in more diverse communities. All environments at all annotation levels examined were more nested than expected randomly, with nestedness higher at higher annotation levels (*p*-value < 0.0001 for all comparisons, from two-tailed tests). **e**, As in **c** but with each environment at EMPO 2 shown separately, with samples colored by EMPO 4.

**Extended Data Fig. 6:**
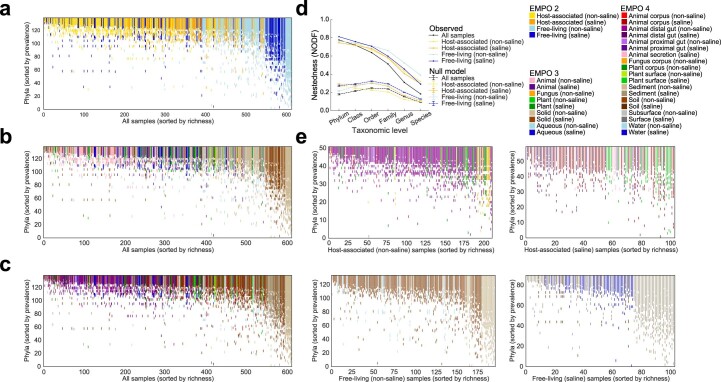
Nestedness of community composition based on microbial taxa. Presence-absence of phyla across samples, with phyla (rows) sorted by prevalence and samples (columns, *n =* 612) sorted by richness. With increasing sample richness, phyla tended to be gained but not lost (SES = 91.86, *p*-value < 0.0001 vs. a null model; nestedness measure based on overlap and decreasing fills [NODF] statistic = 0.78). Samples are colored by EMPO 2. **b**, As in **a** but with samples colored by EMPO 3. **c**, As in **a** but with samples colored by EMPO 4. **d**, Nestedness as a function of taxonomic level, from phylum to species, across all samples and within environments based on EMPO 2. Also shown are median null model NODF scores (± s.d.) for all samples, as well as samples at each level of EMPO 2. NODF measures the average fraction of taxa from less diverse communities that occur in more diverse communities. All environments at all taxonomic levels examined were more nested than expected randomly, with nestedness higher at higher taxonomic levels (*p*-value < 0.0001 for all comparisons, from two-tailed tests). **e**, As in **c** but with each environment at EMPO 2 shown separately, with samples colored by EMPO 4.

In the absence of complete turnover in metabolites and microbial taxa across environments, apparent in the overlap of clusters representing different habitats in our ordinations (Fig. 3c,d), we quantified nestedness. Nestedness describes the degree to which features in one environment are nested subsets of another environment, and can provide insight into community assembly dynamics^1,53^. We found that samples were significantly nested on the basis of both metabolites (Extended Data Fig. 5) and microbial taxa (Extended Data Fig. 6), and that certain environments were consistently nested within others, although this pattern varied between datasets. For example, on the basis of microbial taxa, we observed host-associated samples to be nested within free-living ones (Extended Data Fig. 6a); however, the opposite was true for metabolites (Extended Data Fig. 5a). When considering host association and salinity (that is, EMPO 2) for metabolites, free-living samples were more nested than host-associated ones, and within each group, non-saline samples were more nested than saline ones (Extended Data Fig. 5d). This pattern remained consistent when describing metabolites at the superclass, class and molecular formula levels (Extended Data Fig. 5d). Patterns of nestedness were less consistent across taxonomic levels when based on microbial taxa, although non-saline, free-living samples were the most nested across the family, genus and species levels (Extended Data Fig. 6d). When considering all environments together (that is, for EMPO 3 and 4), we observed stronger patterns of nestedness among environments for microbial taxa (Extended Data Fig. 5b,c) vs metabolites (Extended Data Fig. 6b,c). However, we observed that patterns of nestedness were somewhat similar between microbial taxa and metabolites for host-associated environments, except for plant surfaces (Extended Data Figs. 5e and 6e).

### Metabolites and microbes distinguish habitats

**Fig. 4:**
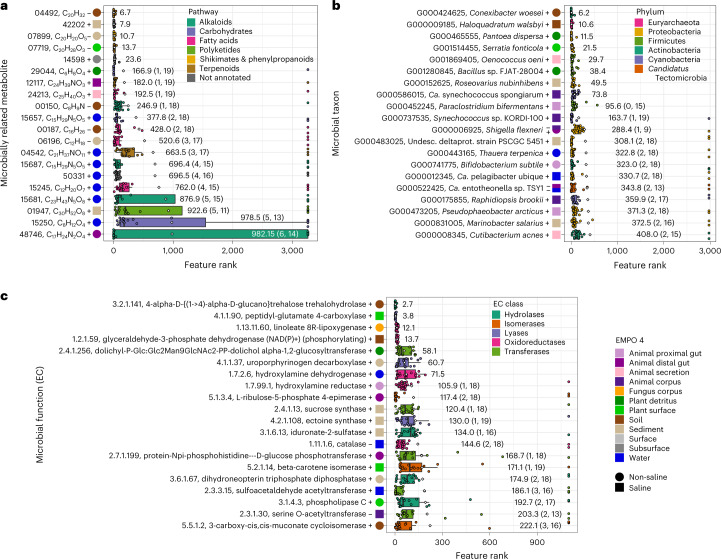
Machine-learning analysis of microbially related metabolites, microbial taxa and microbial functions, highlighting the top 20 most impactful features for each dataset. **a**, The top 20 most impactful microbially related metabolites. Features are coloured by metabolite pathway. Metabolites in bold font are those also identified as important in differential abundance analysis (Supplementary Table 3). **b**, The top 20 most impactful microbial taxa (that is, OGUs). Taxa are coloured by phylum. **c**, The top 20 most impactful microbial functions (that is, KEGG ECs). Boxplots are in the style of Tukey, where the centre line indicates the median, lower and upper hinges the first and third quartiles, respectively, and each whisker is 1.5× IQR from its respective hinge. Enzymes are coloured by class. For all features, ranks are based on impacts derived from SHAP values. Associations with environments are indicated, where + indicates a positive association and – indicates a negative association based on feature abundances. Diamonds and values to the right of boxes indicate means. Values in parentheses indicate (1) the number of iterations (*n* = 20) in which a feature had no impact and (2) the number of iterations in which the reported association was observed, for cases in which values were <20. Environments are described by the Earth Microbiome Project Ontology (EMPO 4).

**Extended Data Fig. 7:**
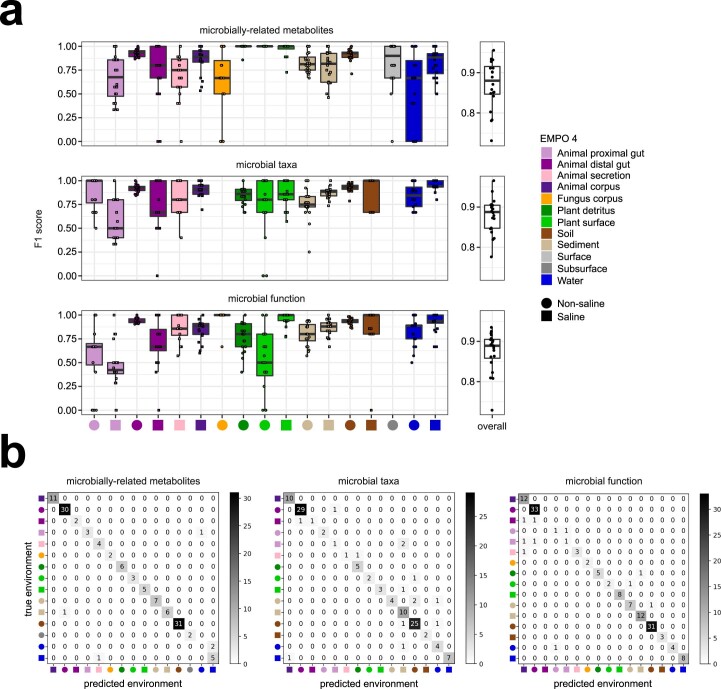
Machine-learning analysis of microbially-related metabolites, microbial taxa, and microbial functions, highlighting per-environment classification performance. **a**, The F1 score (that is, which considers precision and recall) for each environment as well as overall across all environments. For each data layer, every environment is represented by *n* = 20 iterations. **b**, Confusion matrices for each data layer highlighting which pairs of environments are confused. Boxplots are in the style of Tukey, where the center line indicates the median, lower and upper hinges the first- and third quartiles, respectively, and each whisker 1.5 x the interquartile range (IQR) from its respective hinge. For all analyses, environments are described by the Earth Microbiome Project Ontology (EMPO 4).

On the basis of the strong relationships among metabolites, microbes and the environment, we next tested the hypothesis that specific metabolites, microbial taxa or microbial functional products (that is, enzymes) could be used to classify samples among environments. Importantly, features useful in classifying samples among habitats can be used as indicators, which can be useful for detecting certain environmental states, environmental change, or in predicting the diversity of other features. Using a machine-learning classifier (see Online Methods), we identified specific metabolites that classified samples among environments with 88.0% overall accuracy (Fig. 4a, Extended Data Fig. 7a, and Supplementary Fig. 1 and Table 7). After ranking all metabolites on the basis of their impact in distinguishing environments, we found those top ranked to include a diterpenoid negatively associated with non-saline soils (C_20_H_32_, ID: 04492), an undescribed metabolite positively associated with marine sediments (ID: 42202) and a lignan negatively associated with freshwater sediments (C_20_H_20_O_5_, ID: 07899) (Fig. 4a and Supplementary Table 7). Among the top 20 ranked metabolites with annotations, the majority were alkaloids, fatty acids or terpenoids, with terpenoids being the most impactful among the top 10 ranked metabolites, including the most highly ranked one (Fig. 4a and Supplementary Table 7).

We also found strong support among methods for the importance of particular metabolites in distinguishing environments. For example, the undescribed metabolite positively associated with marine sediments (that is, ID: 42202) and one fatty acid—a monoacylglycerol (that is, ID: 42202)—revealed as useful in classification in this analysis also stood out in our analysis of differential abundance (Fig. 4a, and Supplementary Tables 3 and 7). Similarly, distinct analytical approaches identified specific metabolites as particularly important for distinguishing aquatic samples (that is, one glycerolipid, C_28_H_58_O_15_, ID: 14665 and one pseudoalkaloid, C_18_H_22_N_7_O_5_, ID: 14675), non-saline plant surface samples (that is, one chalcone, C_13_H_10_O, ID: 4949) and non-saline animal distal gut samples (that is, one cholane steroid, C_24_H_38_O_4_, ID: 2552 and one prenyl quinone monoterpenoid, C_29_H_46_O_2_, ID: 22299) (Fig. 3c, and Supplementary Tables 3 and 4).

Using the same machine-learning approach on our metagenomic sequence data, we identified specific microbial taxa and microbial functional products (that is, enzymes) useful in classifying samples to environments, with 88.8% and 88.9% overall accuracy, respectively (Fig. 4b,c, Extended Data Fig. 7a, and Supplementary Figs. 2 and 3). We observed that the majority of the top 20 ranked microbial taxa with respect to classification performance were Proteobacteria (Fig. 4b). Cyanobacteria, Firmicutes and Actinobacteria were represented by a few members each, and *Candidatus* Tectomicrobia and Euryarchaeota were represented as singletons (Fig. 4b). The most highly ranked taxon, *Conexibacter woesei* (G000424625, Actinobacteria), was positively associated with non-saline soils, and is an early-diverging member of the class Actinobacteria first isolated from temperate forest soil in Italy^54^ (Fig. 4b). Also among the top ranked taxa were *Haloquadratum walsbyi* (Euryarchaeota) positively associated with saline soils, and *Pantoea dispersa* (*Gammaproteobacteria*) positively associated with the detritus of terrestrial plants (Fig. 4b). For microbial functions, we note that the majority of the top 20 most highly ranked enzymes with respect to classification performance were oxidoreductases or transferases, followed by hydrolases, and then isomerases and lyases (Fig. 4c). The most highly ranked enzyme was positively associated with non-saline soils and was a trehalohydrase (enzyme code (EC): 3.2.1.141), an enzyme that binds trehalose, a carbon-source commonly produced by soil inhabitants including plants, invertebrates, bacteria and fungi, with potential roles in symbioses^55^. Also among the most highly ranked enzymes were a glutamate carboxylase (EC: 4.1.1.90) positively associated with the surfaces of marine plants, and a linoleate lipoxygenase (EC: 1.13.11.60) positively associated with lichen thalli (Fig. 4c).

### Metabolite–microbe co-occurrences are habitat-specific

**Extended Data Fig. 8:**
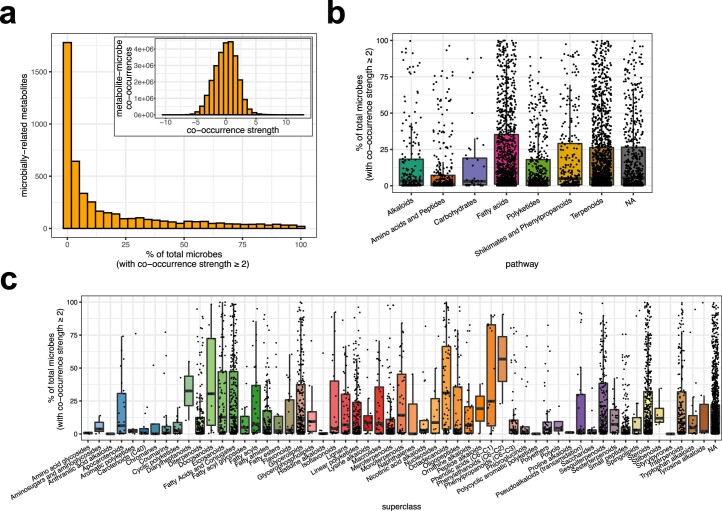
Summary of co-occurrence ranks for microbially-related metabolites. **a**, Distribution of the percentage of microbial taxa for which co-occurrences were strong. Strong co-occurrence was defined as having a co-occurrence strength (that is, rank, or log conditional probability) ≥ 2. The overall distribution of co-occurrence strengths is shown in the inset (*n* = 26,784,120). For values > 0 (*n* = 13,851,755), the minimum = –10.17, maximum = 12.69, mean = 2.40 x 10^-18^, median = 0.08, and mode = 1.22. For values ≥ 2 (*n* = 3,496,639), the minimum = 2.00, maximum = 12.69, mean = 2.87, median = 2.63, and mode = 4.26. **b**, The percentage of microbial taxa for which co-occurrences were strong (that is, ≥ 2), across metabolite pathways. **c**, The percentage of microbial taxa for which co-occurrences were strong (that is, ≥ 2), across metabolite superclasses. For panels **b** and **c**, points were jittered horizontally for clarity, and *n* = 4,765 metabolites. Boxplots are in the style of Tukey, where the center line indicates the median, lower and upper hinges the first- and third quartiles, respectively, and each whisker 1.5 x the interquartile range (IQR) from its respective hinge.

**Extended Data Fig. 9:**
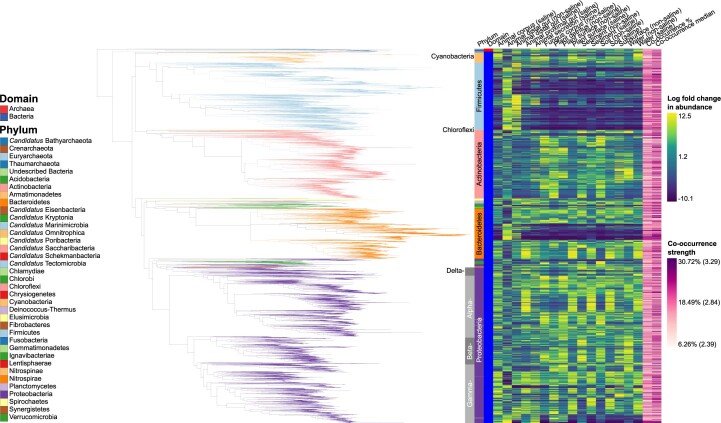
Phylogenetic relationships among microbial taxa highlighting log fold changes in abundance relative to environment, and overall co-occurrences with microbially-related metabolites. Branches are colored by microbial phylum. Annotations include Domain and Phylum level associations (and Class for *Proteobacteria*), heat maps representing log fold changes in relative abundance for each environment (from *songbird*), and heat maps summarizing co-occurrences with microbially-related metabolites (from *mmvec*). Co-occurrence strength indicates (1) the percentage of all microbially-related metabolites for which the co-occurrence rank (that is, log conditional probability) was ≥ 2 (that is, strong), and (2) the median co-occurrence rank value, considering only strong values (in parentheses in the legend).

In addition to exploring relationships between metabolite and microbial diversity, we sought to explicitly quantify metabolite–microbe co-occurrence patterns. Beyond relating metabolites to the microbes that potentially interact with them, certain metabolite–microbe pairs may have stronger associations with the environment than any one feature set alone and may serve as emergent indicators. In particular, we examined associations between metabolites and the environment (for example, Fig. 3a,c) while also considering each metabolite’s co-occurrence with all microbes in the dataset (Extended Data Fig. 1). In that regard, we first generated metabolite–microbe co-occurrences learned from both LC–MS/MS- and shotgun metagenomic profiles across all samples, for a cross-section of 6,501 microbially related metabolites and 4,120 microbial taxa (Extended Data Figs. 8 and 9). Whereas most metabolites co-occurred with at least a few microbes, few metabolites were found to co-occur with many microbes (Extended Data Fig. 8a). The distribution of co-occurrences was not heavily shifted towards any particular pathway (Extended Data Fig. 8b); however, certain superclasses exhibited co-occurrences with many microbes, including diarylheptanoids and phenylethanoids (C6-C3) (Extended Data Fig. 8c). Similarly for microbes, co-occurrences with metabolites were not heavily skewed towards particular phyla, although specific clades were enriched, such as the most recently diverged members of the Bacteroidetes (Extended Data Fig. 9). In contrast to their co-occurrences with metabolites, changes in microbial abundances with respect to the environment appear to be phylogenetically conserved, and correlated with salinity and association with the animal gut environment (Extended Data Fig. 9).

**Fig. 5:**
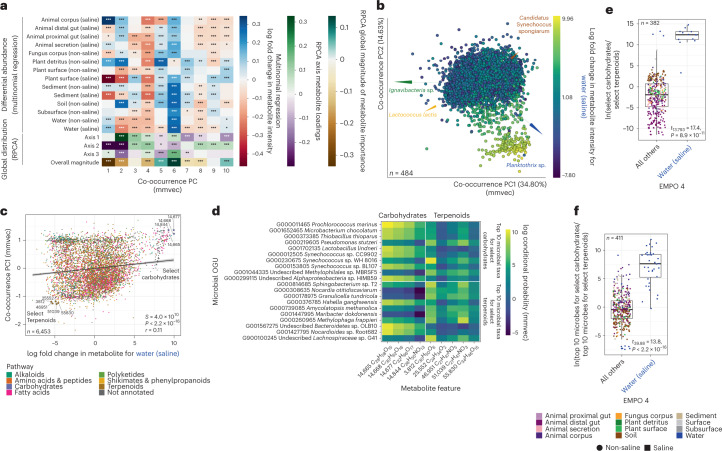
Metabolite–microbe co-occurrences vary across environments. **a**, Correlation between metabolite loadings from the co-occurrence ordination (that is, co-occurrence PCs) and (1) log fold changes in metabolite abundances across environments, (2) metabolite loadings from the ordination in Fig. 3d (that is, Global distribution, axes 1–3) and (3) a vector representing the overall magnitude of microbial taxon abundances from the ordination in Fig. 3d (that is, Global distribution, Overall magnitude). Values are Spearman correlation coefficients. Asterisks indicate significant correlations (*P < 0.05, **P < 0.01, ***P < 0.001). **b**, The relationship between log fold changes in metabolite abundance with respect to ‘Water (non-saline)’ and the first three PCs of the co-occurrence ordination. Points represent metabolites, and the distance between metabolites indicates similarity in their co-occurrences with microbial taxa. Metabolites are coloured on the basis of log fold changes with respect to ‘Water (non-saline)’. Arrows represent specific microbial taxa (colours), distances between arrow tips indicate similarity in their co-occurrence with specific metabolites, and the direction of each arrow indicates which metabolites each microbe co-occurs most strongly with. **c**, The relationship between log fold changes in metabolite abundances with respect to ‘Water (non-saline)’ and loadings for metabolites on PC1 of the co-occurrence ordination. The correlation is one example from **a**. Metabolites are coloured by pathway. Select carbohydrates (excluding glycosides) (the focal group) and select terpenoids (the reference group) are highlighted. **d**, The top 10 co-occurring microbial taxa for all select carbohydrates and all select terpenoids, with a heat map showing co-occurrence strength. **e**, Log-ratio of metabolite intensities for select carbohydrates and select terpenoids. **f**, Log-ratio of abundances of the top 10 microbial taxa associated with select carbohydrates and with select terpenoids. For **e** and **f**, points represent samples, and results from a *t*-test comparing ‘Water (saline)’ vs all other environments are shown. Boxplots are Tukey’s, where the centre indicates the median, lower and upper hinges the first and third quartiles, respectively, and each whisker represents 1.5× IQR from its hinge. For **a**, **c**, **e** and **f**, *P* values are from two-sided tests. For **a** and **c**, *P* values were adjusted using the Benjamini-Hochberg procedure.

**Extended Data Fig. 10:**
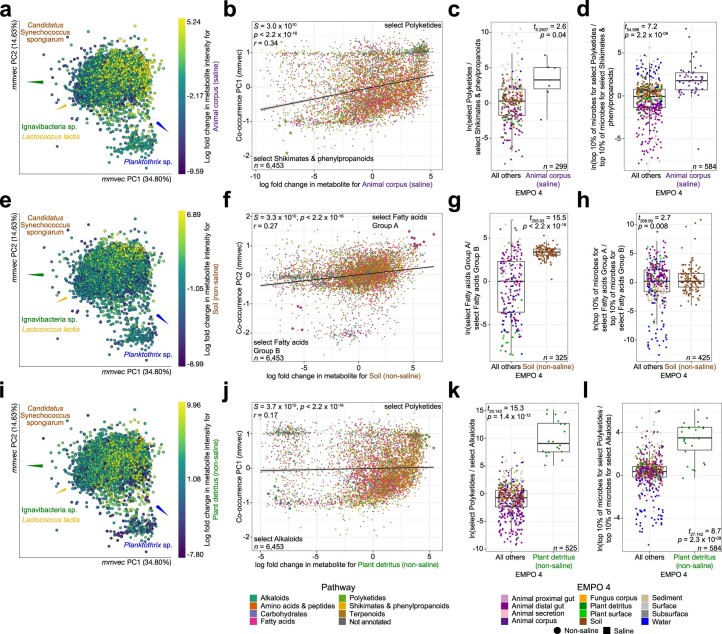
Metabolite-microbe co-occurrences reveal exhibit strong turnover across environments. Results from three environments in addition to ‘Water (saline)’, to highlight differences driven by salinity and host-association: ‘Animal corpus (saline)’, ‘Soil (non-saline)’, and ‘Plant detritus (non-saline’). **a, e, i**, The relationship between log fold changes in abundance for metabolites with respect to the focal environment, and the first three co-occurrence PCs. See Fig. 5 for details. **b, f, j** The relationship between log fold changes in metabolite abundances with respect to the focal environment and loadings for metabolites on PC1 of the co-occurrence ordination. The correlations are examples from Fig. 5a. Metabolites are colored by pathway. Select features representing the focal group and reference group are highlighted, and are described along with the top ten co-occurring microbial taxa for each group in Supplementary Table S5. *P*-values are from two-tailed tests, and were adjusted for multiple comparisons using the Benjamini Hochberg procedure. **c, g, k**, Log-ratio of metabolite intensities for select focal group features and select reference group features with respect to the focal environment. **d, h, l**, Log-ratio of abundances of the top ten microbial taxa associated with focal group metabolites and with reference group metabolites, with respect to the focal environment (see Supplementary Table S5). For panels **c**, **d**, **g**, **h**, **k**, and **l**, points represent samples, and results from a two-sided *t*-test comparing the focal vs. all other environments are shown. Boxplots are Tukey’s, where the center indicates the median, lower and upper hinges the first- and third quartiles, respectively, and each whisker 1.5 x the interquartile range (IQR) from its hinge.

Next, using metabolite–metabolite distances based on co-occurrence profiles considering all microbes, we ordinated metabolites in microbe space. We then examined correlations between metabolite loadings on the principal coordinates of that co-occurrence ordination and (1) log fold changes of metabolites across environments (for example, Fig. 3a) and (2) distributions of metabolites across all samples (that is, loadings and overall magnitude from ordination of all samples) (Fig. 3c), and found strong relationships with each (Fig. 5a). In particular, the abundances of microbially related metabolites in plant surface (saline), sediment (saline) and aquatic samples (that is, those from water) had strong correlations with microbe–metabolite co-occurrences (Fig. 5a). Focusing on seawater (that is, Water (saline)), we visualized the correlation between metabolite loadings on PC1 of the co-occurrence ordination, which represent differences based on co-occurrences with microbes (Fig. 5b), and log fold changes in metabolite abundances with respect to seawater (Fig. 5c). In this space, features with high values for both vectors should be associated with the same microbes and also highly abundant in the ocean, whereas features with low values for both vectors should be associated with the same microbes and have low-to-zero abundance in the ocean (Fig. 5c). Focusing on one group of carbohydrates (excluding glycosides) and one group of terpenoids (Fig. 5c,d), we found significant differences in their intensities in seawater vs all other environments (Fig. 5e), as well as in the abundances of their top co-occurring microbial taxa (Fig. 5f). Importantly, by relying on our metabolite intensity data, this result validates patterns identified in our analyses of differential abundance across environments and co-occurrence with microbial taxa. We used this same approach to explore metabolite–microbe co-occurrences specific to other environments (Extended Data Fig. 10 and Supplementary Table 5), further revealing strong turnover in metabolite–microbe co-occurrences across habitats.

### Correlations with amplicon sequence data and GC–MS data

To begin to explore the additional data generated for EMP500 samples, including GC–MS and amplicon sequence data (that is, bacterial and archaeal 16S and full-length rRNA operon, eukaryotic 18S, fungal ITS), we compared sample–sample distances (that is, beta-diversity) between each pair of datasets. Beyond providing insight into how certain community data are related, strong correlations between datasets may indicate similarity in the structuring of features among samples or habitats. Importantly, we found further support for a strong relationship between microbially related metabolites and microbial taxa (LC–MS/MS vs 16S; *r* = 0.27, *P* = 0.001) (Table 1). The relationships between the metabolomics data (that is, LC–MS/MS or GC–MS) and sequence data from eukaryotes (that is, 18S or ITS) were weaker (for example, LC–MS/MS vs ITS; *r* = 0.07, *P* = 0.006) (Table 1). The weakest relationships were between sequence data from Bacteria and Archaea (that is, 16S or shotgun metagenomics) and sequence data from eukaryotes (that is, 18S or ITS) (for example, shotgun metagenomics for taxa vs. 18S; *r* = –0.002, *P* = 0.9) (Table 1). The strongest relationships were between different layers of sequence data from Bacteria and Archaea (Table 1). For example, correlations between 16S rRNA profiles and those from full-length rRNA operons had *r* = 0.55 (*P* = 0.001), and 16S vs shotgun metagenomics (taxa) had *r* = 0.51 (*P* = 0.001) (Table 1). These results highlight the strong relationship between metabolic profiles and microbial taxonomic composition across habitats spanning the globe.

## Discussion

Here we discuss some of the caveats and limitations of our study, and further highlight how our approach advances understanding of microbial community dynamics and functional diversity. Due to their extensive nature, we provide additional important points of discussion as Supplementary Information. We begin by recognizing that certain environments included in EMPO are represented here by only a handful of samples (Fig. 1) and/or a single sample set (Supplementary Table 1), and note that we had to exclude them from some of our analyses due to low representation (for example, machine learning and co-occurrence analyses). We recommend that future efforts focus on additional sampling of these environments to further generalize our findings to those habitats. Similarly, we hope to expand sampling geographically to broaden our scope of inference, as many important environments and locations could not be included here (or, indeed, in the EMP’s 27,000-sample dataset^1^). We also note that the inherent design of the EMP (that is, crowd-sourced samples from experts in respective fields) prevented us from explicitly exploring causation with respect to the environment in our analysis, and thus our findings are based largely on observations and correlations among feature sets and associated metadata.

In our example analysis, we explored whether every metabolite is everywhere but the environment selects (that is, the Baas Becking hypothesis^42,43^, but for microbially related metabolites). Whereas we interpret our findings as strong evidence that every metabolite is everywhere but the environment selects, our study was not designed to address this hypothesis explicitly, and further evidence is needed to support this hypothesis. For example, features at abundances below the detection limit of our approach could not be considered here, but may alter our view of these patterns. Similarly, although input sample volumes were normalized as best as possible, they may influence estimates of alpha-diversity, and the values reported here probably exhibit some error in part due to this influence. We also identified metabolite–microbe co-occurrences, and note that our approach for characterizing co-occurrences, ‘mmvec’^56^, does not currently allow for controlling for covariates and this may influence results. However, in our analysis we were able to include EMPO as a variable, which we designed to account for variation among environments that may not be captured by available metadata.

Here we described patterns of turnover, nestedness and co-occurrence of metabolites and microbes across a diverse set of environments while addressing ecological questions surrounding the distribution of metabolites and their relationships with microbial taxonomic and functional diversity. One outstanding question in microbial ecology asks how microbial taxon profiles can be integrated with functional ones^57^. Here, in addition to describing microbial taxa, their functions and their metabolites, we explicitly tested for metabolite–microbe co-occurrences and explored how they relate to the environment, for which we have outlined our approach (Extended Data Fig. 1). Our analysis provides insight into biological processes including microbial community assembly and links microbial taxonomic profiles with metabolism and functional diversity (that is, enzymes) at planetary scale. Our work provides an initial view of how microbially related metabolites are structured with respect to factors including host association, salinity and the presence of certain microbes (Figs. 3 and 5). Importantly, we identified the most abundant and highly ranked pathway representing the metabolites best able to distinguish environments to be terpenoids^58^, highlighting the importance of this group of metabolites in distinguishing Earth’s environments (Fig. 4a and Supplementary Table 7).

We acknowledge that previous studies describing microbial taxa and function using globally distributed sample sets, such as for the human gut, soils and the ocean, have shown that both can vary across locations^59–62^. Similarly, studies examining metabolite profiles across changes in microbial community composition, or environmental stress such as from heat, have shown variation associated with either^20,21^ or both^23^. Furthermore, among previous multi-omics studies combining metagenomics with metatranscriptomics, metaproteomics and/or metabolomics, some of which have shown the correlation between data layers to vary across sites, the majority are focused on a single environment^63–73^. Here we performed multi-omics integration of a dataset encompassing a diversity of environmental sample types representing several habitats, generated using standardized methods allowing for robust meta-analysis with data from other studies using the same approach.

Our approach illustrates that recent advances in computational annotation tools offer a powerful toolbox to interpret untargeted metabolomics data^41^. We anticipate that parallel advances in metagenomic sequencing, genome assembly and genome mining will improve the discovery and classification of functional products from among microbes and provide additional insight into these findings. By following standardized methods available on GitHub and making this dataset publicly available in Qiita and GNPS, this study will serve as an important resource for continued collaborative investigations. In the same manner, the development of optimized instrumentation and computational methods for metabolomics will expand the depth of metabolites surveyed in microbiome studies.

## Methods

### Dataset description

#### Sample collection

Our research complies with all relevant ethical regulations following policies at the University of California, San Diego (UCSD). Animal samples that were sequenced were not collected at UCSD and are not for vertebrate animals research at UCSD following the UCSD Institutional Animal Care and Use Committee (IACUC). Samples were contributed by 34 principal investigators of the Earth Microbiome Project 500 (EMP500) Consortium and are samples from studies at their respective institutions (Supplementary Table 1). Relevant permits and ethics information for each parent study are described in the ‘Permits for sample collection’ section below. Samples were contributed as distinct sets referred to here as studies, where each study represented a single environment (for example, terrestrial plant detritus). To achieve more even coverage across microbial environments, we devised an ontology of sample types (microbial environments), the EMP Ontology (EMPO) (http://earthmicrobiome.org/protocols-and-standards/empo/)^1^, and selected samples to fill out EMPO categories as broadly as possible. EMPO recognizes strong gradients structuring microbial communities globally, and thus classifies microbial environments (level 4) on the basis of host association (level 1), salinity (level 2), host kingdom (if host-associated) or phase (if free-living) (level 3) (Fig. 1a). As we anticipated previously^1^, we have updated the number of levels as well as states therein for EMPO (Fig. 1b) on the basis of an important additional salinity gradient observed among host-associated samples when considering the previously unreported shotgun metagenomic and metabolomic data generated here (Fig. 3c,d). We note that although we were able to acquire samples for all EMPO categories, some categories are represented by a single study.

Samples were collected following the Earth Microbiome Project sample submission guide^50^. Briefly, samples were collected fresh, split into 10 aliquots and then frozen, or alternatively collected and frozen, and subsequently split into 10 aliquots with minimal perturbation. Aliquot size was sufficient to yield 10–100 ng genomic DNA (approximately 10^7^–10^8^ cells). To leave samples amenable to chemical characterization (metabolomics), buffers or solutions for sample preservation (for example, RNAlater) were avoided. Ethanol (50–95%) was allowed as it is compatible with LC–MS/MS although it should also be avoided if possible.

Sampling guidance was tailored for four general sample types: bulk unaltered (for example, soil, sediment, faeces), bulk fractionated (for example, sponges, corals, turbid water), swabs (for example, biofilms) and filters. Bulk unaltered samples were split fresh (or frozen), sampled into 10 pre-labelled 2 ml screw-cap bead beater tubes (Sarstedt, 72.694.005 or similar), ideally with at least 200 mg biomass, and flash frozen in liquid nitrogen (if possible). Bulk fractionated samples were fractionated as appropriate for the sample type, split into 10 pre-labelled 2 ml screw-cap bead beater tubes, ideally with at least 200 mg biomass, and flash frozen in liquid nitrogen (if possible). Swabs were collected as 10 replicate swabs using 5 BD SWUBE dual cotton swabs with wooden stick and screw cap (281130). Filters were collected as 10 replicate filters (47 mm diameter, 0.2 um pore size, polyethersulfone (preferred) or hydrophilic PTFE filters), placed in pre-labelled 2 ml screw-cap bead beater tubes, and flash frozen in liquid nitrogen (if possible). All sample types were stored at –80 °C if possible, otherwise –20 °C.

To track the provenance of sample aliquots, we employed a QR coding scheme. Labels were affixed to aliquot tubes before shipping when possible. QR codes had the format ‘name.99.s003.a05’, where ‘name’ is the PI name, ‘99’ is the study ID, ‘s003’ is the sample number and ‘a05’ is the aliquot number. QR codes (version 2, 25 pixels × 25 pixels) were printed on 1.125’ × 0.75’ rectangular and 0.437’ circular cap Cryogenic Direct Thermal labels (GA International, DFP-70) using a Zebra model GK420d printer and ZebraDesigner Pro 3 software for Windows. After receipt but before aliquots were stored in freezers, QR codes were scanned into a sample inventory spreadsheet using a QR scanner.

#### Sample metadata

Environmental metadata were collected for all samples on the basis of the EMP Metadata Guide, which combines guidance from the Genomics Standards Consortium MIxS (Minimum Information about any Sequence) standard^74^ and the Qiita Database (https://qiita.ucsd.edu)^51^. The metadata guide provides templates and instructions for each MIxS environmental package (that is, sample type). Relevant information describing each PI submission, or study, was organized into a separate study metadata file (Supplementary Table 1).

### Metabolomics

#### LC–MS/MS sample extraction and preparation

To profile metabolites among all samples, we used LC–MS/MS, a versatile method that detects tens of thousands of metabolites in biological samples. All solvents and reactants used were LC–MS grade. To maximize the biomass extracted from each sample, the samples were prepared depending on their sampling method (for example, bulk, swabs, filter and controls). The bulk samples were transferred into a microcentrifuge tube (polypropylene, PP) and dissolved in 7:3 MeOH:H_2_O using a volume varying from 600 µl to 1.5 ml, depending on the amounts of sample available, and homogenized in a tissue lyser (QIAGEN) at 25 Hz for 5 min. Then, the tubes were centrifuged at 2,000 × *g* for 15 min, and the supernatant was collected in a 96-well plate (PP). For swabs, the swabs were transferred into a 96-well plate (PP) and dissolved in 1.0 ml of 9:1 ethanol:H_2_O. The prepared plates were sonicated for 30 min, and after 12 h at 4 °C, the swabs were removed from the wells. The filter samples were dissolved in 1.5 ml of 7:3 MeOH:H_2_O in microcentrifuge tubes (PP) and sonicated for 30 min. After 12 h at 4 °C, the filters were removed from the tubes. The tubes were centrifuged at 2,000 × *g* for 15 min, and the supernatants were transferred to 96-well plates (PP). The process control samples (bags, filters and tubes) were prepared by adding 3.0 ml of 2:8 MeOH:H_2_O and recovering 1.5 ml after 2 min. After the extraction process, all sample plates were dried with a vacuum concentrator and subjected to solid phase extraction (SPE). SPE was used to remove salts that could reduce ionization efficiency during mass spectrometry analysis, as well as the most polar and non-polar compounds (for example, waxes) that cannot be analysed efficiently by reversed-phase chromatography. The protocol was as follows: the samples (in plates) were dissolved in 300 µl of 7:3 MeOH:H_2_O and put in an ultrasound bath for 20 min. SPE was performed with SPE plates (Oasis HLB, hydrophilic-lipophilic-balance, 30 mg with particle sizes of 30 µm). The SPE beds were activated by priming them with 100% MeOH, and equilibrated with 100% H_2_O. The samples were loaded on the SPE beds, and 100% H_2_O was used as wash solvent (600 µl). The eluted washing solution was discarded, as it contains salts and very polar metabolites that subsequent metabolomics analysis is not designed for. The sample elution was carried out sequentially with 7:3 MeOH:H_2_O (600 µl) and 100% MeOH (600 µl). The obtained plates were dried with a vacuum concentrator. For mass spectrometry analysis, the samples were resuspended in 130 µl of 7:3 MeOH:H_2_O containing 0.2 µM of amitriptyline as an internal standard. The plates were centrifuged at 30 × *g* for 15 min at 4 °C. Samples (100 µl) were transferred into new 96-well plates (PP) for mass spectrometry analysis.

#### LC–MS/MS sample analysis

The extracted samples were analysed by ultra-high performance liquid chromatography (UHPLC, Vanquish, Thermo Fisher) coupled to a quadrupole-Orbitrap mass spectrometer (Q Exactive, Thermo Fisher) operated in data-dependent acquisition mode (LC–MS/MS in DDA mode). Chromatographic separation was performed using a Kinetex C_18_ 1.7 µm (Phenomenex), 100 Å pore size, 2.1 mm (internal diameter) × 50 mm (length) column with a C_18_ guard cartridge (Phenomenex). The column was maintained at 40 °C. The mobile phase was composed of a mixture of (A) water with 0.1% formic acid (v/v) and (B) acetonitrile with 0.1% formic acid. Chromatographic elution method was set as follows: 0.00–1.00 min, isocratic 5% B; 1.00–9.00 min, gradient from 5% to 100% B; 9.00–11.00 min, isocratic 100% B; followed by equilibration 11.00–11.50 min, gradient from 100% to 5% B; 11.50–12.50 min, isocratic 5% B. The flow rate was set to 0.5 ml min^−1^.

The UHPLC was interfaced to the orbitrap using a heated electrospray ionization source with the following parameters: ionization mode, positive; spray voltage, +3,496.2 V; heater temperature, 363.90 °C; capillary temperature, 377.50 °C; S-lens RF, 60 arbitrary units (a.u.); sheath gas flow rate, 60.19 a.u.; and auxiliary gas flow rate, 20.00 a.u. The MS^1^ scans were acquired at a resolution (at *m*/*z* 200) of 35,000 in the *m*/*z* 100–1500 range, and the fragmentation spectra (MS^2^) scans at a resolution of 17,500 from 0 to 12.5 min. The automatic gain control target and maximum injection time were set at 1.0 × 10^6^ and 160 ms for MS^1^ scans, and set at 5.0 × 10^5^ and 220 ms for MS^2^ scans, respectively. Up to three MS^2^ scans in data-dependent mode (Top 3) were acquired for the most abundant ions per MS^1^ scans using the apex trigger mode (4–15 s), dynamic exclusion (11 s) and automatic isotope exclusion. The starting value for MS^2^ was m/z 50. Higher-energy collision induced dissociation (HCD) was performed with a normalized collision energy of 20, 30 and 40 eV in stepped mode. The major background ions originating from the SPE were excluded manually from the MS^2^ acquisition. Analyses were randomized within plate and blank samples analysed every 20 injections. A quality control mix sample assembled from 20 random samples across the sample types was injected at the beginning, the middle and the end of each plate sequence. The chromatographic shift observed throughout the batch was estimated as less than 2 s, and the relative standard deviation of ion intensity was 15% per replicate.

#### LC–MS/MS data processing

The mass spectrometry data were centroided and converted from the proprietary format (.raw) to the *m*/*z* extensible markup language format (.mzML) using ProteoWizard (ver. 3.0.19, MSConvert tool)^75^. The mzML files were then processed with MZmine 2 toolbox^76^ using the ion-identity networking modules^77^ that allow advanced detection for adduct/isotopologue annotations. The MZmine processing was performed on Ubuntu 18.04 LTS 64-bits workstation (Intel Xeon E5-2637, 3.5 GHz, 8 cores, 64 Gb of RAM) and took ~3 d. The MZmine project, the MZmine batch file (.XML format) and results files (.MGF and .CSV) are available in the MassIVE dataset MSV000083475. The MZmine batch file contains all the parameters used during the processing. In brief, feature detection and deconvolution was performed with the ADAP chromatogram builder^78^ and local minimum search algorithm. The isotopologues were regrouped and the features (peaks) were aligned across samples. The aligned peak list was gap filled and only peaks with an associated fragmentation spectrum and occurring in a minimum of three files were conserved. Peak shape correlation analysis grouped peaks originating from the same molecule and annotated adduct/isotopologue with ion-identity networking^77^. Finally, the feature quantification table results (.CSV) and spectral information (.MGF) were exported with the GNPS module for feature-based molecular networking analysis on GNPS^79^ and with SIRIUS export modules.

#### LC–MS/MS data annotation

The results files of MZmine (.MGF and .CSV files) were uploaded to GNPS (http://gnps.ucsd.edu)^52^ and analysed with the feature-based molecular networking workflow^79^. Spectral library matching was performed against public fragmentation spectra (MS^2^) spectral libraries on GNPS and the NIST17 library.

For the additional annotation of small peptides, we used the DEREPLICATOR tools available on GNPS^80,81^. We then used SIRIUS^82^ (v. 4.4.25, headless, Linux) to systematically annotate the MS^2^ spectra. Molecular formulae were computed with the SIRIUS module by matching the experimental and predicted isotopic patterns^83^, and from fragmentation trees analysis^84^ of MS^2^. Molecular formula prediction was refined with the ZODIAC module using Gibbs sampling^85^ on the fragmentation spectra (chimeric spectra or those with poor fragmentation were excluded). In silico structure annotation using structures from biodatabase was done with CSI:FingerID^86^. Systematic class annotations were obtained with CANOPUS^41^ and used the NPClassifier ontology^87^.

The parameters for SIRIUS tools were set as follows, for SIRIUS: molecular formula candidates retained, 80; molecular formula database, ALL; maximum precursor ion *m*/*z* computed, 750; profile, orbitrap; *m*/*z* maximum deviation, 10 ppm; ions annotated with MZmine were prioritized and other ions were considered (that is, [M+H3N+H]+, [M+H]+, [M+K]+, [M+Na]+, [M+H-H2O]+, [M+H-H4O2]+, [M+NH4]+); for ZODIAC: the features were split into 10 random subsets for lower computational burden and computed separately with the following parameters: threshold filter, 0.9; minimum local connections, 0; for CSI:FingerID: *m*/*z* maximum deviation, 10 ppm; and biological database, BIO.

To establish putative microbially related secondary metabolites, we collected annotations from spectral library matching and the DEREPLICATOR+ tools and queried them against the largest microbial metabolite reference databases (Natural Products Atlas^88^ and MIBiG^89^). Molecular networking^79^ was then used to propagate the annotation of microbially related secondary metabolites throughout all molecular families (that is, the network component).

#### LC–MS/MS data analysis

We combined the annotation results from the different tools described above to create a comprehensive metadata file describing each metabolite feature observed. Using that information, we generated a feature-table including only secondary metabolite features determined to be microbially related. We then excluded very low-intensity features introduced to certain samples during the gap-filling step described above. These features were identified on the basis of presence in negative controls that were universal to all sample types (that is, bulk, filter and swab) and by their relatively low per-sample intensity values. Finally, we excluded features present in positive controls for sampling devices specific to each sample type (that is, bulk, filter or swab). The final feature-table included 618 samples and 6,588 putative microbially related secondary metabolite features that were used for subsequent analysis.

We used QIIME 2’s^90^ (v2020.6) ‘diversity’ plugin to quantify alpha-diversity (that is, feature richness) for each sample and ‘deicode’^91^ to quantify beta-diversity (that is, robust Aitchison distances, which are robust to both sparsity and compositionality in the data) between each pair of samples. We parameterized our robust Aitchison principal components analysis (RPCA)^91^ to exclude samples with fewer than 500 features and features present in fewer than 10% of samples. We used the ‘taxa’ plugin to quantify the relative abundance of microbially related secondary metabolite pathways and superclasses (that is, on the basis of NPClassifier) within each environment (that is, for each level of EMPO 4), and ‘songbird’ v1.0.4^92^ to identify sets of microbially related secondary metabolites whose abundances were associated with certain environments. We parameterized our ‘songbird’ model as follows: epochs, 1,000,000; differential prior, 0.5; learning rate, 1.0 × 10^−5^; summary interval, 2; batch size, 400; minimum sample count, 0; and training on 80% of samples at each level of EMPO 4 using ‘Animal distal gut (non-saline)’ as the reference environment. Environments with fewer than 10 samples were excluded to optimize model training (that is, ‘Animal corpus (non-saline)’, ‘Animal proximal gut (non-saline)’, ‘Surface (saline)’). The output from ‘songbird’ includes a rank value for each metabolite in every environment, which represents the log fold change for a given metabolite in a given environment^92^. We compared log fold changes for each metabolite from this run to those from (1) a replicate run using the same reference environment and (2) a run using a distinct reference environment: ‘Water (saline)’. We found strong Spearman correlations in both cases (Supplementary Table 8), and therefore focused on results from the original run using ‘Animal distal gut (non-saline)’ as the reference environment, as it has previously been shown to be relatively unique among other habitats. In addition to summarizing the top 10 metabolites for each environment (Supplementary Table 3), we used the log fold change values in our multi-omics analyses described below.

We used the RPCA biplot and QIIME 2’s^90^ EMPeror^93^ to visualize differences in composition among samples, as well as the association with samples of the 25 most influential microbially related secondary metabolite features (that is, those with the largest magnitude across the first three principal component loadings). We tested for significant differences in metabolite composition across all levels of EMPO using PERMANOVA implemented with QIIME 2’s ‘diversity’ plugin^90^ and using our robust Aitchison distance matrix as input. In parallel, we used the differential abundance results from ‘songbird’ described above to identify specific microbially related secondary metabolite pathways and superclasses that varied strongly across environments. We then went back to our metabolite feature-table to visualize differences in the relative abundances of those pathways and superclasses within each environment by first selecting features and calculating log-ratios using ‘qurro’^94^, and then plotting using the ‘ggplot2’ package^95^ in R^96^ v4.0.0. We tested for significant differences in relative abundances across environments using Kruskal–Wallis tests implemented with the base ‘stats’ package in R^96^.

#### GC–MS sample extraction and preparation

To profile volatile small molecules among all samples in addition to what was captured with LC–MS/MS, we used gas chromatography coupled with mass spectrometry (GC–MS). All solvents and reactants were GC–MS grade. Two protocols were used for sample extraction, one for the 105 soil samples and a second for the 356 faecal and sediment samples that were treated as biosafety level 2. The 105 soil samples were received at the Pacific Northwest National Laboratory and processed as follows. Each soil sample (1 g) was weighed into microcentrifuge tubes (Biopur Safe-Lock, 2.0 ml, Eppendorf). H_2_O (1 ml) and one scoop (~0.5 g) of a 1:1 (v/v) mixture of garnet (0.15 mm, Omni International) and stainless steel (0.9–2.0 mm blend, Next Advance) beads and one 3 mm stainless steel bead (Qiagen) were added to each tube. Samples were homogenized in a tissue lyser (Qiagen) for 3 min at 30 Hz and transferred into 15 ml polypropylene tubes (Olympus, Genesee Scientific). Ice-cold water (1 ml) was used to rinse the smaller tube and combined into the 15 ml tube. Chloroform:methanol (10 ml, 2:1 v/v) was added and samples were rotated at 4 °C for 10 min, followed by cooling at −70 °C for 10 min and centrifuging at 150 × *g* for 10 min to separate phases. The top and bottom layers were combined into 40 ml glass vials and dried using a vacuum concentrator. Chloroform:methanol (1 ml, 2:1) was added to each large glass vial and the sample was transferred into 1.5 ml tubes and centrifuged at 1,300 × *g*. The supernatant was transferred into glass vials and dried for derivatization.

The remaining 356 samples received from UCSD that included faecal and sediment samples were processed as follows: 100 µl of each sample was transferred to a 2 ml microcentrifuge tube using a scoop (MSP01, Next Advance). The final volume of the sample was brought to 1.5 ml, ensuring that the solvent ratio is 3:8:4 H_2_O:CHCl_3_:MeOH by adding the appropriate volumes of H_2_O, MeOH and CHCl_3_. After transfer, one 3 mm stainless steel bead (QIAGEN), 400 µl methanol and 300 µl H_2_O were added to each tube and the samples were vortexed for 30 s. Then, 800 µl chloroform was added and samples were vortexed for 30 s. After centrifuging at 150 × *g* for 10 min to separate phases, the top and bottom layers were combined in a vial and dried for derivatization.

The samples were derivatized for GC–MS analysis as follows: 20 µl of a methoxyamine solution in pyridine (30 mg ml^−1^) was added to the sample vial and vortexed for 30 s. A bath sonicator was used to ensure that the sample was completely dissolved. Samples were incubated at 37 °C for 1.5 h while shaking at 1,000 r.p.m. *N*-methyl-*N*-trimethylsilyltrifluoroacetamide (80 µl) and 1% trimethylchlorosilane solution was added and samples were vortexed for 10 s, followed by incubation at 37 °C for 30 min, with 1,000 r.p.m. shaking. The samples were then transferred into a vial with an insert.

An Agilent 7890A gas chromatograph coupled with a single quadrupole 5975C mass spectrometer (Agilent) and an HP-5MS column (30 m × 0.25 mm × 0.25 μm; Agilent) was used for untargeted analysis. Samples (1 μl) were injected in splitless mode, and the helium gas flow rate was determined by the Agilent Retention Time Locking function on the basis of analysis of deuterated myristic acid (Agilent). The injection port temperature was held at 250 °C throughout the analysis. The GC oven was held at 60 °C for 1 min after injection, and the temperature was then increased to 325 °C at a rate of 10 °C min^−1^, followed by a 10 min hold at 325 °C. Data were collected over the mass range of *m*/*z* 50–600. A mixture of FAMEs (C8–C28) was analysed each day with the samples for retention index alignment purposes during subsequent data analysis.

#### GC–MS data processing and annotation

The data were converted from vendor’s format to the .mzML format and processed using GNPS GC–MS data analysis workflow (https://gnps.ucsd.edu)^97^. The compounds were identified by matching experimental spectra to the public libraries available at GNPS, as well as NIST 17 and Wiley libraries. The data are publicly available at the MassIVE depository (https://massive.ucsd.edu); dataset ID: MSV000083743. The GNPS deconvolution is available in GNPS (https://gnps.ucsd.edu/ProteoSAFe/status.jsp?task=d5c5135a59eb48779216615e8d5cb3ac), as is the library search (https://gnps.ucsd.edu/ProteoSAFe/status.jsp?task=59b20fc8381f4ee6b79d35034de81d86).

#### GC–MS data analysis

For multi-omics analyses including GC–MS data, we first removed noisy (that is, suspected background contaminants and artifacts) features by excluding those with balance scores <50%. Balance scores describe compositional consistency of deconvoluted spectra across the dataset, where high values indicate reproducible spectral patterns and thus high-quality spectra. We then used QIIME 2’s ‘deicode’^91^ plugin to estimate beta-diversity for each dataset using robust Aitchison distances. The final feature-table for GC–MS beta-diversity analysis included 460 samples and 216 features.

### Metagenomics

#### DNA extraction

For each round of DNA extractions described below for both amplicon and shotgun metagenomic sequencing, a single aliquot of each sample was processed for DNA extraction. DNA was extracted following the EMP 96-sample, magnetic bead-based DNA extraction protocol^98^ following refs. ^99–101^ and using the QIAGEN MagAttract PowerSoil DNA KF kit (384-sample) (that is, optimized for KingFisher, 27100-EP). Importantly, material from each sample was added to a unique bead tube (containing garnet beads) for single-tube lysis, which has been shown to reduce sample-to-sample contamination common in plate-based extractions^101^. For bulk samples, 0.1–0.25 g of material was added to each well; for filtered samples, one entire filter was added to each well; for swabbed samples, one swab head was added to each well. The lysis solution was dissolved at 60 °C before addition to each tube, then capped tubes were incubated at 65 °C for 10 min before mechanical lysis at 6,000 r.p.m. for 20 min using a MagNA lyser (Roche). Lysate from each bead tube was then randomly assigned and added to wells of a 96-well plate, and then cleaned-up using the KingFisher Flex system (Thermo Fisher). Resulting DNA was stored at –20 °C for sequencing. We note that whereas QIAGEN does not offer a ‘hybrid’ extraction kit allowing for single-tube lysis and plate-based clean-up, the Thermo MagMAX Microbiome Ultra kit does, and was recently shown to be comparable to the EMP protocol used here^102^.

#### Amplicon sequencing

We generated amplicon sequence data for variable region four (V4) of the bacterial and archaeal 16S rRNA gene, variable region nine (V9) of the eukaryotic 18S rRNA gene, and the fungal internal transcribed spacer one (ITS1). For amplifying and sequencing all targets, we used a low-cost, miniaturized (that is, 5 µl volume), high-throughput (384-sample) amplicon library preparation method implementing the Echo 550 acoustic liquid handler (Beckman Coulter)^103^. The same protocol was modified with different primer sets and PCR cycling parameters depending on the target. Two rounds of DNA extraction and sequencing were performed for each target to obtain greater coverage per sample. For a subset of 500 samples, we also generated high-quality sequence data for full-length bacterial rRNA operons following a previously published protocol^104^, which is briefly outlined below.

The protocol for 16S is outlined fully in ref. ^105^. To target the V4 region, we used the primers 515F (Parada) (5’-GTGYCAGCMGCCGCGGTAA-3’) and 806R (Apprill) (5’-GGACTACNVGGGTWTCTAAT-3’). These primers are updated from the original EMP 16S-V4 primer sequences^106,107^ to (1) remove bias against Crenarchaeota/Thaumarchaeota^108^ and the marine freshwater clade SAR11 (Alphaproteobacteria)^109^, and (2) enable the use of various reverse primer constructs (for example, the V4-V5 region using the reverse primer 926R^110^) by moving the barcode/index to the forward primer^108^. We note that while we previously named these updated primers ‘515FB’ and ‘806RB’ to distinguish them from the original primers, the ‘B’ may be misinterpreted to indicate ‘Barcode’. To avoid ambiguity, we now use the original names suffixed with the lead author name (that is, ‘515F (Parada)’, ‘806R (Apprill)’ and ‘926R (Quince)’). We highly recommend to always check the primer sequence in addition to the primer name. For Qiita users, studies with ‘library_construction_protocol’ as ‘515f/806rbc’ used the original primers, whereas ‘515fbc/806r’ indicates use of updated primers, where ‘bc’ refers to the location of the barcode.

To facilitate sequencing on Illumina platforms, the following primer constructs were used to integrate adapter sequences during amplification^106,107,111^. For the barcoded forward primer, constructs included (5’ to 3’): the 5’ Illumina adapter (AATGATACGGCGACCACCGAGATCTACACGCT), a Golay barcode (12 bp variable sequence), a forward primer pad (TATGGTAATT), a forward primer linker (GT) and the forward primer (515F (Parada)) (GTGYCAGCMGCCGCGGTAA). For the reverse primer, constructs included (5’ to 3’): the reverse complement of 3’ Illumina adapter (CAAGCAGAAGACGGCATACGAGAT), a reverse primer pad (AGTCAGCCAG), a reverse primer linker (CC) and the reverse primer (806R (Apprill)) (GGACTACNVGGGTWTCTAAT).

For each 25 µl reaction, we combined 13 µl PCR-grade water (Sigma W3500, or QIAGEN 17000-10), 10 µl Platinum Hot Start PCR master mix (2X) (Thermo Fisher, 13000014), 0.5 µl of each primer (10 µM) and 1 µl of template DNA. The final concentration of the master mix in each 1X reaction was 0.8X and that of each primer was 0.2 µM. Cycling parameters for a 384-well thermal cycler were as follows: 94 °C for 3 min; 35 cycles of 94 °C for 1 min, 50 °C for 1 min and 72 °C for 105 s; and 72 °C for 10 min. For a 96-well thermal cycler, we recommend the following: 94 °C for 3 min; 35 cycles of 94 °C for 45 s, 50 °C for 1 min and 72 °C for 90 s; and 72 °C for 10 min.

We amplified each sample in triplicate (that is, each sample was amplified in three replicate 25 µl reactions) and pooled products from replicate reactions for each sample into a single volume (75 µl). We visualized expected products between 300–350 bp on agarose gels, and note that while low-biomass samples may yield no visible bands, instruments such as a Bioanalyzer or TapeStation (Agilent) can be used to confirm amplification. We quantified amplicons using the Quant-iT PicoGreen dsDNA Assay kit (Thermo Fisher, P11496) following the manufacturer’s instructions. To pool samples, we combined an equal amount of product from each sample (240 ng) into a single tube and cleaned the pool using the UltraClean PCR Clean-Up kit (QIAGEN, 12596-4) following the manufacturer’s instructions. We checked DNA quality using a Nanodrop (Thermo Fisher), confirming that A260/A280 ratios were between 1.8–2.0.

For sequencing, the following primer constructs were used. Read 1 constructs included (5’ to 3’): a forward primer pad (TATGGTAATT), a forward primer linker (GT) and the forward primer (515F (Parada)) (GTGYCAGCMGCCGCGGTAA). Read 2 constructs included (5’ to 3’): a reverse primer pad (AGTCAGCCAG), a reverse primer linker (CC) and the reverse primer (806R (Apprill)) (GGACTACNVGGGTWTCTAAT). The index primer sequence was AATGATACGGCGACCACCGAGATCTACACGCT, which we highlight as having an extra GCT at the 3’ end compared to Illumina’s index primer sequence, to increase the melting temperature for read 1 during sequencing.

The protocol for 18S is outlined fully in ref. ^112^. To target variable region nine (V9), we used the primers 1391f (5’-GTACACACCGCCCGTC-3’) and EukBr (5’-TGATCCTTCTGCAGGTTCACCTAC-3’). These primers are based on those of ref. ^113,114^ and are designed for use with Illumina platforms. The forward primer is a universal small-subunit primer, whereas the reverse primer favours eukaryotes but with mismatches can bind and amplify Bacteria and Archaea. In addition to deviations from the 16S protocol above with respect to primer construct sequences and PCR cycling parameters, we included a blocking primer that reduces amplification of vertebrate host DNA for host-associated samples, on the basis of the strategy outlined in ref. ^115^. We note that the blocking primer is particularly useful for host-associated samples with a low biomass of non-host eukaryotic DNA.

The following primer constructs were used to integrate adapter sequences during amplification. For the barcoded forward primer, constructs included (5’ to 3’): the 5’ Illumina adapter (AATGATACGGCGACCACCGAGATCTACAC), a forward primer pad (TATCGCCGTT), a forward primer linker (CG) and the forward primer (Illumina_Euk_1391f) (GTACACACCGCCCGTC). For the reverse primer, constructs included (5’ to 3’): The reverse complement of 3’ Illumina adapter (CAAGCAGAAGACGGCATACGAGAT), a Golay barcode (12 bp variable sequence), a reverse primer pad (AGTCAGTCAG), a reverse primer linker (CA) and the reverse primer (806R (Apprill)) (TGATCCTTCTGCAGGTTCACCTAC). The construct for the blocking primer is as such and is formatted for ordering from IDT: ‘GCCCGTCGCTACTACCGATTGG/ideoxyI//ideoxyI//ideoxyI//ideoxyI//ideoxyI/TTAGTGAGGCCCT/3SpC3/’.

Reaction mixtures without the blocking primer (that is, those for non-vertebrate hosts or free-living sample types as defined by EMPO) were prepared as described for 16S. For reactions including the blocking primer, we combined 9 µl PCR-grade water, 10 µl master mix, 0.5 µl of each primer (10 µM), 4 µl of blocking primer (10 µM) and 1 µl of template DNA. The final concentration of the master mix in each 1X reaction was 0.8X, that of each primer was 0.2 µM and that of the blocking primer was 1.6 µM. Without blocking primers, cycling parameters for a 384-well thermal cycler were as follows: 94 °C for 3 min; 35 cycles of 94 °C for 45 s, 57 °C for 1 min and 72 °C for 90 s; and 72 °C for 10 min. With blocking primers, cycling parameters for a 384-well thermal cycler were as follows: 94 °C for 3 min; 35 cycles of 94 °C for 45 s, 65 °C for 15 s, 57 °C for 30 s and 72 °C for 90 s; and 72 °C for 10 min. Expected bands ranged between 210–310 bp.

For sequencing, the following primer constructs were used. Read 1 constructs (Euk_illumina_read1_seq_primer) included (5’ to 3’): a forward primer pad (TATCGCCGTT), a forward primer linker (CG) and the forward primer (1391f) (GTACACACCGCCCGTC). Read 2 constructs (Euk_illumina_read2_seq_primer) included (5’ to 3’): a reverse primer pad (AGTCAGTCAG), a reverse primer linker (CA) and the reverse primer (EukBr) (TGATCCTTCTGCAGGTTCACCTAC). The index primer construct (Euk_illumina_index_seq_primer) included (5’ to 3’): the reverse complement of the reverse primer (EukBr) (GTAGGTGAACCTGCAGAAGGATCA), the reverse complement of the reverse primer linker (TG) and the reverse complement of the reverse primer pad (CTGACTGACT).

The protocol for ITS is outlined fully in ref. ^116^. To target the fungal internal transcribed spacer (ITS1), we used the primers ITS1f (5’-CTTGGTCATTTAGAGGAAGTAA-3’) and ITS2 (5’-GCTGCGTTCTTCATCGATGC-3’). These primers are based on those of ref. ^117^, and we note that primer ITS1f used here binds 38 bp upstream of ITS1 reported in that study.

The following primer constructs were used to integrate adapter sequences during amplification. For the barcoded forward primer, constructs included (5’ to 3’): the 5’ Illumina adapter (AATGATACGGCGACCACCGAGATCTACAC), a forward primer linker (GG) and the forward primer (ITS1f) (CTTGGTCATTTAGAGGAAGTAA). For the reverse primer, constructs included (5’ to 3’): the reverse complement of 3’ Illumina adapter (CAAGCAGAAGACGGCATACGAGAT), a Golay barcode (12 bp variable sequence), a reverse primer linker (CG) and the reverse primer (ITS2) (GCTGCGTTCTTCATCGATGC).

Reaction mixtures were prepared as described for 16S. Cycling parameters for a 384-well thermal cycler were as follows: 94 °C for 1 min; 35 cycles of 94 °C for 30 s, 52 °C for 30 s and 68 °C for 30 ; and 68 °C for 10 min. Expected bands ranged between 250–600 bp^118,119^.

For sequencing, the following primer constructs were used. Read 1 sequencing primer constructs included (5’ to 3’): a forward primer segment (TTGGTCATTTAGAGGAAGTAA) and a region extending into the amplicon (AAGTCGTAACAAGGTTTCC). Read 2 sequencing primer constructs included (5’ to 3’): a reverse primer segment (CGTTCTTCATCGATGC) and a region extending into the amplicon (VAGARCCAAGAGATC). The index sequencing primer construct included (5’ to 3’): the reverse complement of the region extending into the amplicon (TCTC), the reverse complement of the reverse primer (GCATCGATGAAGAACGCAGC) and the reverse complement of the linker (CG).

The protocol for generating bacterial full-length rRNA operon data is described in ref. ^104^. The method uses a unique molecular identifier (UMI) strategy to remove PCR errors and chimeras, resulting in a mean error rate of 0.0007% and a chimera rate of 0.02% of the final amplicon data. Briefly, the bacterial rRNA operons were targeted with an initial PCR using tailed versions of 27f (AGRGTTYGATYMTGGCTCAG)^120^ and 2490r (GACGGGCGGTGWGTRCA)^121^. The primer tails contained synthetic priming sites and 18-bp-long patterned UMIs (NNNYRNNNYRNNNYRNNN). The PCR reaction (50 µl) contained 1–2 ng DNA template, 1 U Platinum SuperFi DNA Polymerase High Fidelity (Thermo Fisher) and a final concentration of 1× SuperFi buffer, 0.2 mM of each deoxynucleotide triphosphate, and 500 nM of each tailed 27f and tailed 2490r. The PCR cycling parameters consisted of an initial denaturation (3 min at 95 °C) and two cycles of denaturation (30 s at 95 °C), annealing (30 s at 55 °C) and extension (6 min at 72 °C). The PCR product was purified using a custom bead purification protocol ‘SPRI size selection protocol for >1.5–2 kb DNA fragments’ (Oxford Nanopore Technologies). The resulting product consists of uniquely tagged rRNA operon amplicons. The uniquely tagged rRNA operons were amplified in a second PCR, where the reaction (100 µl) contained 2 U Platinum SuperFi DNA Polymerase High Fidelity (Thermo Fisher) and a final concentration of 1X SuperFi buffer, 0.2 mM of each dNTP, and 500 nM of each forward and reverse synthetic primer targeting the tailed primers from above. The PCR cycling parameters consisted of an initial denaturation (3 min at 95 °C) and then 25–35 cycles of denaturation (15 s at 95 °C), annealing (30 s at 60 °C) and extension (6 min at 72 °C), followed by final extension (5 min at 72 °C). The PCR product was purified using the custom bead purification protocol above. Batches of 25 amplicon libraries were barcoded and sent for PacBio Sequel II library preparation and sequencing (Sequel II SMRT Cell 8M and 30 h collection time) at the DNA Sequencing Center at Brigham Young University. Circular consensus sequencing (CCS) reads were generated using CCS v.3.4.1 (https://github.com/PacificBiosciences/ccs) using default settings. UMI consensus sequences were generated using the longread_umi pipeline (https://github.com/SorenKarst/longread_umi) with the following command: longread_umi pacbio_pipeline -d ccs_reads.fq -o out_dir -m 3500 -M 6000 -s 60 -e 60 -f CAAGCAGAAGACGGCATACGAGAT -F AGRGTTYGATYMTGGCTCAG -r AATGATACGGCGACCACCGAGATC -R CGACATCGAGGTGCCAAAC -U ‘0.75;1.5;2;0’ -c 2.

#### Amplicon data analysis

For multi-omics analyses including amplicon sequence data, we processed each dataset for comparison of beta-diversity. For all amplicon data except that for bacterial full-length rRNA amplicons, raw sequence data were converted from bcl to fastq, and then multiplexed files for each sequencing run uploaded as separate preparations to Qiita (study: 13114).

For each 16S sequencing run, in Qiita, data were demultiplexed, trimmed to 150 bp and denoised using Deblur^122^ to generate a feature-table of sub-operational taxonomic units (sOTUs) per sample, using default parameters. We then exported feature-tables and denoised sequences from each sequencing run, used QIIME 2’s ‘feature-table’ plugin to merge feature-tables and denoised reads across sequencing runs, and placed all denoised reads into the GreenGenes 13_8 phylogeny^123^ via fragment insertion using QIIME 2’s^90^ SATé-Enabled Phylogenetic Placement (SEPP)^124^ plugin to produce a phylogeny for diversity analyses. To allow for phylogenetically informed diversity analyses, reads not placed during SEPP (that is, 513 sOTUs, 0.1% of all sOTUs) were removed from the merged feature-table. We then used QIIME 2’s ‘feature-table’ plugin to exclude singleton sOTUs and rarefy the data to 5,000 reads per sample. Rarefaction depths for all amplicon analyses were chosen to best normalize sampling effort per sample while maintaining ≥75% of samples representative of Earth’s environments, and also to maintain consistency with the analyses from EMP release 1. We then used QIIME 2’s^90^ ‘diversity’ plugin to estimate alpha-diversity (that is, sOTU richness) and beta-diversity (that is, unweighted UniFrac distances). The final feature-table for 16S beta-diversity analysis included 681 samples and 93,260 features. We performed a comparative analysis of the data including and excluding the reads not placed during SEPP, and note that both alpha-diversity (that is, sOTU richness) and beta-diversity (that is, sample–sample RPCA distances) were highly correlated between datasets (Spearman *r* = 1.0) (Supplementary Fig. 5). We thus proceeded with the SEPP-filtered dataset and used phylogenetically informed diversity metrics where applicable.

For 18S data, we used QIIME 2’s^90^ ‘demux’ plugin’s ‘emp-paired’ method^125,126^ to first demultiplex each sequencing run, and then the ‘cutadapt’ plugin’s^127^ ‘trim-paired’ method to trim sequencing primers from reads. We then exported trimmed reads, concatenated R1 and R2 read files per sample, and denoised reads using Deblur’s^122,128^ ‘workflow’ with default settings, trimming reads to 90 bp, and taking the ‘all.biom’ and ‘all.seqs’ output, for each sequencing run. We then used QIIME 2’s ‘feature-table’ plugin to merge feature-tables and denoised sequences across sequencing runs, and then the ‘feature-classifier’ plugin’s ‘classify-sklearn’ method to classify taxonomy for each sOTU via pre-fitted machine-learning classifiers^129^ and the SILVA 138 reference database^130^. We then used QIIME 2’s^90^ ‘feature-table’ plugin to exclude reads assigned to bacteria and archaea, singleton sOTUs and samples with a total frequency of <5,500 reads, and the ‘deicode’^91^ plugin to estimate beta-diversity for each dataset using robust Aitchison distances^91^. The final feature-table for 18S beta-diversity analysis included 461 samples and 14,839 features.

For fungal ITS data, we used QIIME 2^90^ to generate and merge feature-tables and denoised sequences across sequencing runs, as for 18S data but trimming reads to 150 bp. We then classified taxonomy for each sOTU as for 18S data, but using the UNITE 9 reference database^131^. We then used QIIME 2’s ‘feature-table’ plugin to exclude singleton sOTUs and samples with a total frequency of <500 reads, and the ‘deicode’^91^ plugin to estimate beta-diversity for each dataset using robust Aitchison distances^91^. The final feature-table for fungal ITS beta-diversity analysis included 500 samples and 10,966 features.

For full-length rRNA operon data, per-sample fasta files were reformatted for importing to QIIME 2 as ‘SampleData[Sequences]’ (that is, with each header as ‘>{sample_identifier}_{sequence_identifier}’), concatenated into a single fasta file and imported. We then used QIIME 2’s ‘vsearch’ plugin^132^ to dereplicate sequences and then cluster them at 65% similarity (that is, due to rapid evolution at bacterial ITS regions). The 65% OTU feature-table had 365 samples and 285 features. The concatenated fasta file and 65% OTU feature-table were uploaded to Qiita as distinct preparations (study: 13114). We then used QIIME 2’s^90^ ‘feature-table’ plugin to exclude singleton OTUs and samples with a total frequency of <500 reads, and the ‘deicode’^91^ plugin to estimate beta-diversity for each dataset using robust Aitchison distances^91^. The final feature-table for full-length rRNA operon beta-diversity analysis included 242 samples and 196 features.

#### Shotgun metagenomic sequencing

One round of DNA extraction was performed as above for shotgun metagenomic sequencing. Sequencing libraries were prepared using a high-throughput version of the HyperPlus library chemistry (Kapa Biosystems) miniaturized to approximately 1:10 reagent volume and optimized for nanolitre-scale liquid-handling robotics^133^. An exhaustive step-by-step protocol and accompanying software can be found in ref. ^133^. Briefly, DNA from each sample was transferred to a 384-well plate and quantified using the Quant-iT PicoGreen dsDNA Assay kit (P7589, Thermo Fisher), and then normalized to 5 ng in 3.5 µl of molecular-grade water using an Echo 550 acoustic liquid-handling robot (Labcyte). For library preparation, reagents for each step (that is, fragmentation, end repair and A-tailing, ligation and PCR) were added at 1:10 the recommended volumes using a Mosquito HTS micropipetting robot (SPT Labtech). Fragmentation was performed at 37 °C for 20 min and A-tailing at 65 °C for 30 min.

Sequencing adapters and barcode indices were added in two steps^134^. First, the Mosquito HTS robot was used to add universal adapter ‘stub’ adapters and ligase mix to the end-repaired DNA, and the ligation reaction performed for 20 °C for 1 h. Adapter-ligated DNA was then cleaned-up using AMPure XP magnetic beads and a BlueCat purification robot (BlueCat Bio) by adding 7.5 µl magnetic bead solution to the total sample volume, washing twice with 70% ethanol and resuspending in 7 µl molecular-grade water. Then, the Echo 550 robot was used to add individual i7 and i5 indices to adapter-ligated samples without repeating any barcodes, and iterate the assignment of i7 to i5 indices to minimize repeating unique i7:i5 pairs. Cleaned adapter-ligated DNA was then amplified by adding 4.5 µl of each sample to 5.5 µl PCR master mix and running for 15 cycles, and then purified again using magnetic beads and the BlueCat robot. Each sample was eluted into 10 µl water, and then transferred to a 384-well plate using the Mosquito HTS robot. Each library was quantified using qPCR and then pooled to equal molar fractions using the Echo 550 robot. The final pool was sequenced at Illumina on a NovaSeq6000 using S2 flow cells and 2 × 150 bp chemistry (Illumina). To increase sequence coverage for certain samples, libraries were re-pooled and a second sequencing run performed as above.

#### Shotgun data analysis

Raw sequence data were converted from bcl to fastq and demultiplexed to produce per-sample fastq files. The mean sequencing depth was 7,580,347 ± 7.82 × 10^13^ reads per sample. We processed raw reads with Atropos (v1.1.24)^135^ to trim universal adapter sequences, poly-G tails introduced by the NovaSeq instrument (that is, from use of two-colour chemistry) and low-quality bases from reads. Atropos parameters included poly-G trimming (nextseq-trim=30), inclusion of ambiguous bases (match-read-wildcards), a maximum error rate for adapter matching (error-rate=0.1, default), removal of low-quality bases at 3’ and 5’ ends before adapter removal (quality-cutoff=15), a maximum error rate for adapter matching (insert-match-error-rate=0.2, default), discarding of short trimmed reads (minimum-length=100) and discarding of paired reads if even one fails filtering (pair-filter=any). Trimmed reads were then mapped to the Web of Life database of microbial genomes (release 1)^136^ using bowtie2 v2.3.2^137^ in very-sensitive mode to produce alignments that were used for taxonomic and exploratory functional analysis of microbial communities. Bowtie2 settings included maximum and minimum mismatch penalties (mp=[1,1]), a penalty for ambiguities (np=1; default), read and reference gap open- and extend penalties (rdg=[0,1], rfg=[0,1]), a minimum alignment score for an alignment to be considered valid (score-min=[L,0,−0.05]), a defined number of distinct valid alignments (k=16), and the suppression of SAM records for unaligned reads, as well as SAM headers (no-unal, no-hd). The Web of Life database is particularly attractive as it includes a phylogeny that can be used for diversity analyses, and was curated to represent phylogenetic breadth of Bacteria and Archaea^136^, ideal for analyses across diverse environments. We compared mapping to the Web of Life to Rep200, a curated database of NCBI representative and reference microbial genomes (that is, corresponding to RefSeq release 200, released 14 May 2020) and found little difference across environments (Supplementary Fig. 6). We therefore chose the Web of Life as it allows for phylogenetically informed analyses.

For taxonomic analysis, we generated a feature-table of counts of operational genomic units (OGUs) for each sample using a reference-based approach. We chose this method over the de novo or reference-free approach, as the latter uses assembly/clustering to deconvolute short reads into larger sequence units; the reference-free approach allows for the direct observation of the actual organisms in the community, but alone does not allow their meaningful characterization^6^. Reference-based approaches use reference sequences from described organisms, allowing us to find the closest matches and use them to describe the taxa in a community^6^. This strategy is advantageous as results are not dependent on the samples included and it is less difficult because sequences can more easily be aligned to a reference vs assembled into MAGS^138,139^. Most importantly, it allows for comparisons of results across samples and studies, therefore representing a standardized method. Specifically, we used Woltka’s v0.1.4^140^ ‘classify’ function, with per-genome alignments and default parameters. Woltka’s default normal mode is such that for one query sequence mapped to *k* genomes, each genome receives a count of 1/*k*. To permit examination of rare taxa across environments, no genomes were excluded. For diversity analyses, to best normalize sampling effort per sample while maintaining ≥75% of samples representative of Earth’s environments, we rarefied the OGU feature-table to 6,550 reads per sample. The final feature-table for analyses of shotgun metagenomic taxonomic diversity included 612 samples and 8,692 OGUs.

For alpha-diversity, we quantified three metrics, in part to see which had the strongest correlations with microbially related metabolite richness. We used the R package ‘geiger’^141^ to quantify weighted Faith’s PD for each sample following the method of Swensen^142^. We used QIIME 2’s ‘diversity’ plugin^90^ to quantify richness and Faith’s PD (that is, unweighted), as well as beta-diversity (that is, using weighted UniFrac distance) between each pair of samples. We performed PERMANOVA on that distance matrix to test for significant differences in microbial community composition across the various levels of EMPO, and verified that differences were robust across sampling depths spanning three orders of magnitude (Supplementary Table 9). We then used principal coordinates analysis (PCoA) and EMPeror^93^ to visualize differences in microbial community composition among samples. We used ‘songbird’^92^ to identify sets of microbial taxa whose abundances were associated with certain environments, and parameterized our songbird model as above for our LC–MS/MS data. We then mapped the differential abundance results from songbird onto a phylogeny representing all microbial taxa using ‘empress’^143^ to visualize phylogenetic relationships related to log fold changes in abundance relative to specific environments.

For the functional analysis, we initially generated two sets of annotations for comparison of read mapping across environments. First, we generated a feature-table of counts of Gene Ontology (GO) terms (that is, for biological process, molecular function and cellular compartment) for each sample using Woltka’s ‘collapse’ function, inputting per-gene alignments and with default parameters for mapping to GO terms through MetaCyc. For subsequent analysis, we used QIIME 2’s^90^ ‘feature-table’ plugin to exclude singleton features and rarefy the data to 5,000 sequences per sample. The final feature-table included 517 samples and 3,776 features (that is, GO terms). We also generated a feature-table of counts of KEGG^144–146^ EC features (that is, enzymes) for each sample using PRROMenade^147^. Trimmed, quality-controlled reads were mapped to the PRROMenade index of bacterial and viral protein domains via the IBM Functional Genomics Platform^148^ following ref. ^149^, searching for maximal exact matches with a length ≥11 amino acids and retaining samples with ≥10,000 annotated reads (that is, summed across R1 and R2 read files). Annotated read counts were pushed to leaf level nodes in the four-level EC hierarchy (for example, EC 1.2.3.4). For diversity analysis, we used QIIME 2’s^90^ ‘feature-table’ plugin to exclude singleton features and samples with fewer than 150,000 reads. The final feature-table included 616 samples (representing 18 environments) and 1,250 enzymes (that is, KEGG ECs). We performed a comparative analysis comparing the Woltka GO-term analysis and the PRROMenade KEGG EC analysis, and found PRROMenade to more efficiently map reads across the majority of environments (Supplementary Fig. 4). We therefore proceeded with our analysis of microbial functions using PRROMenade. With that table, we used QIIME 2’s ‘deicode’^91^ plugin to estimate beta-diversity for each dataset using robust Aitchison distances^91^ and EMPeror^93^ to visualize differences in microbial community composition among samples. We then performed PERMANOVA as above to test for significant differences in microbial functional composition across the various levels of EMPO.

#### Nestedness analysis of metabolites and microbial taxa

As our analysis of turnover (replacement) of microbial taxa suggested a degree of nestedness (gain or loss of taxa promoting differences in richness) among environments in line with previous observations based on EMP 16S release 1, we tested for nestedness in our shotgun metagenomics data for microbial taxa. We used the NODF statistic^150^ to quantify nestedness on the basis of the degree to which less diverse communities are subsets of more diverse communities, which we quantified at each major taxonomic level from phylum to species. We used the rarefied feature-table described above and a null model (that is, equiprobable rows, fixed columns) for assessing observed values of NODF, which we considered at each taxonomic level, and for all of the samples and each subset of the samples at EMPO 2. To compute standardized effect sizes and *P* values for significance, we used simulated results (*n* = 10,000 iterations) to find the expectation and variance of the NODF statistic under the null model. Standardized effect sizes were large (>90).

### Multi-omics

#### Alpha-diversity correlations

Using the alpha-diversity metrics for LC–MS/MS (that is, richness) and shotgun metagenomic taxonomic data (that is, richness, unweighted Faith’s PD and weighted Faith’s PD), we performed correlation analysis to better understand relationships therein. We used the function ‘multilevel’ available in the R package ‘correlation’^151^ to perform Spearman correlations for each environment (that is, based on EMPO 4), treating study (that is, the variable representing distinct PI submissions of samples) as a random effect and adjusting for multiple comparisons using the Benjamini-Hochberg correction. We performed additional correlations with our shotgun metagenomics data rarefied to sampling depths across three orders of magnitude, and confirmed that patterns observed with our focal sampling depth of 6,550 are robust, although loss of samples at higher sampling depths results in reduced effects (Supplementary Table 10).

#### Machine-learning analyses

To better understand community composition of microbes and metabolites across environments and specifically which features are predictive of certain habitats, we performed machine learning. For analyses of LC–MS/MS and shotgun metagenomic taxonomic and functional data, additional samples were filtered from the feature-tables noted previously to exclude environments with relatively low sample representation (that is, <9 samples). For the LC–MS/MS feature-table, we excluded samples in the four EMPO environments (that is, ‘Animal corpus (non-saline)’, ‘Animal proximal gut (non-saline)’, ‘Soil (saline)’ and ‘Surface (saline)’). The final feature-table included 605 samples (representing 15 environments) and 6,588 microbially related metabolites. For the shotgun metagenomic feature-table for taxonomic analysis, we excluded samples in four EMPO environments (that is, ‘Animal corpus (non-saline)’, ‘Fungus corpus (non-saline)’, ‘Surface (saline)’ and ‘Subsurface (non-saline)’). The final feature-table included 598 samples (representing 15 environments) and 8,587 microbial taxa (that is, Woltka OGUs). For the shotgun metagenomic feature-table for functional analysis, we used QIIME 2’s^90^ ‘feature-table’ plugin to exclude samples in three EMPO environments (that is, ‘Animal corpus (non-saline)’, ‘Surface (saline)’ and ‘Subsurface (non-saline)’), exclude singleton features and normalize the total count per sample to 10,000 sequences. The final feature-table included 706 samples (representing 16 environments) and 1,133 enzymes (that is, KEGG ECs).

For each feature-table, we trained an auto-AI classifier^152^ with SHAP explanations^153^ and the hyper-tuned XGBoost method^154^ for predicting environments (on the basis of EMPO 4). Each dataset was split into a training set (80%) and a testing set (20%), with similar environmental distributions in each iteration for the classification of samples. We evaluated the predictive performance of each classifier by quantifying accuracy statistics across 20 randomized iterations, and specifically by using resulting confusion matrices to quantify the overall and per-environment precision, recall and F1 score. To identify the most important features contributing to the classification, we examined SHAP explanations, which we used to describe the impact of each feature for prediction. For features with an impact in at least one of 20 iterations examined, we assigned absolute ranks for each feature per iteration, and then assigned final ranks on the basis of the mean of absolute ranks across iterations. For the top 20 ranked features per feature-table, we visualized the environment for which each feature was impactful, as well as the direction of impact. Direction was determined by assessing differences in the mean relative abundances of the focal environment vs all other environments combined. Positive impact indicates that a feature was predictive of the focal environment when it was more abundant there vs the other environments.

#### Metabolite–microbe co-occurrence analysis

To begin to explore co-occurrences between microbes and metabolites across environments, we implemented an approach that generates co-occurrence probabilities between all metabolite and microbial features, clusters metabolites on the basis of their co-occurrence with the microbial community and highlights individual microbial features driving global patterns in metabolite distribution in this space. For co-occurrence analyses of LC–MS/MS metabolites and genomes profiled from shotgun metagenomic data, feature-tables were further filtered to retain only the 434 samples found in both datasets. For the LC–MS/MS feature-table of microbially related secondary metabolites, we excluded 172 samples lacking shotgun metagenomics data, resulting in a final set of 6,501 microbially related metabolites. For the shotgun metagenomics feature-table for taxonomy, we excluded 150 samples lacking LC–MS/MS data, resulting in a final set of 4,120 OGUs.

Specifically, we obtained co-occurrence probabilities and ordinated metabolites in microbial taxon space using ‘mmvec’ v1.0.6, which uses the probabilities (that is, log conditional probabilities, or co-occurrence strength) to predict metabolites on the basis of microbial taxa from neural-network, compositionally robust modelling^56^. The model was trained on 80% of the 434 samples, which were selected to balance environments (that is, EMPO 4), and used the following parameters: epochs, 200; batch size, 165; learning rate, 1.0 × 10^−5^; summary interval, 1; and with ‘equalize-biplot’. For training and testing, we filtered to retain only those features present in at least 10 samples (that is, min-feature-count, 10), and restricted decomposition of the co-occurrence matrix to 10 principal components (PCs) (that is, latent-dim, 10). The model predicting metabolite–microbe co-occurrences was more accurate than one representing a random baseline, with a pseudo-*Q*^2^ value of 0.18, indicating much reduced error during cross-validation.

To relate these metabolite–microbe co-occurrences to the distribution of metabolites across environments, we calculated the Spearman correlation between the loadings of metabolites on each co-occurrence PC vs (1) log fold changes in metabolite abundances for each environment (that is, from ‘songbird’), (2) loadings for metabolites on the first three axes from the ordination corresponding to clustering of samples by environment (that is, from RPCA) and (3) a vector representing the global magnitude of metabolite importance across all three axes from that same ordination. To explicitly highlight metabolite–microbe co-occurrences specific to particular environments, we visualized the relationships between metabolite–microbe co-occurrences and (1) by considering the first three PCs of the co-occurrence ordination (that is, from mmvec) and colouring metabolites by their log fold change values for a focal environment (for example, Fig. 4b, Extended Data Fig. 10 and Supplementary Table 5). Then, focusing on the co-occurrence PC exhibiting the strongest correlation with log fold changes in metabolite abundances with respect to the focal environment, we manually selected one subset of metabolites highly abundant with respect to the focal environment but similar with respect to co-occurrences with microbes (that is, high values on both axes, the focal group of metabolites) and one subset of metabolites lowly abundant with respect to the focal environment but similar with respect to co-occurrences with microbes (that is, low values on both axes, the reference group of metabolites)^155^. Each select group of metabolites was chosen to represent a single pathway. Then, depending on the focal environment, we chose either the top 10 or top 10% of co-occurring microbes (that is, on the basis of co-occurrence strength) for each of the focal and reference groups of metabolites^154^. Finally, we visualized differences in the log-ratio of the focal group to the reference group between the focal environment and all other environments, separately for metabolites and microbes^154^.

#### Mantel correlations between datasets

To explore the relationships between sample–sample distances for any two datasets (for example, LC–MS/MS vs shotgun metagenomics for taxonomy), we used QIIME 2’s ‘diversity’ plugin^90^ to perform Mantel tests on all pairings of the datasets using Spearman correlations. Input distance matrices are those described above for each dataset.

### Statistics and reproducibility

Samples and studies were crowd-sourced to span microbial environments described by EMPO version 1. Before acceptance as an EMP500 study, scientific justification was required (Supplementary Table 1). Sample sizes for each study were determined by each EMP500 PI on the basis of sample availability (that is, no statistical method was used to predetermine sample size, but our sample sizes are similar to those reported in previous studies^59–62,156^). No samples were excluded from analysis, except when inclusion violated assumptions or best practices of statistical tests, which we detail for each method used above. As each sample was split into 10 aliquots, samples from several studies are available for future use. Similarly, as we used standardized protocols and methods throughout from sample collection to data analysis, the results are reproducible. No experiments requiring randomization or blindness were carried out. For each analysis, we used non-parametric statistical tests unless tests for normality and equal variances showed that these assumptions were met.

### Permits for sample collection

For all animal, geological and international sample collection, the proper procedures for sampling, exporting and importing were followed. In accordance with the genetic resource sharing component of the Nagoya Protocol, we have made all sequence data publicly available at NCBI. Here we provide specific statements for sample collection where relevant.

Animal specimens and geological samples used in this study were collected for a range of different parent studies and were contributed to the project at UCSD (Supplementary Table 1). Therefore, based on IACUC policies, this project was not considered vertebrate animal research at UCSD. Here we provide relevant ethical information for samples from parent studies that included animals: studies 9, 18, 63 and 72 include only lower-level invertebrates and are thus exempt from animal use protocol based on IACUC guidelines; study 50 did not require handling of animals; collection for studies 51–53 and 54 was approved by the University of Colorado, Boulder (UCB); collection for study 54 was approved by UCSD (protocols S12219, S09392); study 76 did not require handling of animals; collection for study 81 was approved by UCB (protocol 08-04-AK-01); collection for study 88 was approved by the Animal Experiment Board in Finland (ESAVI/7256/04.10.07/2014).

For samples from Costa Rica, permits were granted by the Institutional Biodiversity Commission of the University of Costa Rica (UCR, resolution number 055-2016) and authorized by the Organization for Tropical Studies (OTS) and the Central Pacific Conservation Area (ACOPAC) of the Ministry of Energy and the Environment (MINAE), Costa Rican government, under UCR project B6-656.

For samples from Ukraine, all procedures were performed in accordance with legal requirements and regulations from Ukrainian authorities (957-i/16/05/2016), and the Animal Experiment Board in Finland (ESAVI/7256/04.10.07/2014). The samples were transported to Finland for research purposes on the basis of the import permission from the Evira (3679/0460/2016).

Samples from Namibia were collected under the Republic of Namibia - Ministry of Mines and Energy permit number ES30246 and transported to South Africa for research purpose with import permit P0067933 from the Department of Agriculture, Forestry and Fisheries of the Republic of South Africa.

For samples from Singapore, permit (No:NP/RP18-086) was granted by National Parks Board (NParks) and sampling was conducting according to stipulations of the permit.

All coral samples were collected by AAUS-certified scientific divers, in accordance with local regulations. Relevant permit numbers are: CITES (PWS2014-AU-002155, 12US784243/9), Great Barrier Reef Marine Park Authority (G12/35236.1, G14/36788.1), Lord Howe Island Marine Park (LHIMP/R/2015/005), New South Wales Department of Primary Industries (P15/0072–1.0, OUT 15/11450), US Fish and Wildlife Service (2015LA1632527, 2015LA1703560), and Western Australia Department of Parks and Wildlife (SF010348, CE004874, ES002315).

### How to access and contribute to the EMP500

All methods and protocols can be accessed at www.earthmicrobiome.org and GitHub (https://github.com/biocore/emp/). All data are available as indicated below. We note that in parallel to future sample collection efforts directed by the EMP Consortium, all projects adhering to the EMP standardized protocols for sample collection and sample processing can be analysed using meta-analyses with the data provided here, and all other data generated by following those protocols. Announcements for future sample collection directed by the EMP500 Consortium will be made via https://earthmicrobiome.org.

### Reporting summary

Further information on research design is available in the Nature Portfolio Reporting Summary linked to this article.

### Supplementary information

Supplementary Information Supplementary Figs 1–6 and Discussion.

Reporting Summary

Supplementary Table Supplementary Tables 1–10.

## Data Availability

The mass spectrometry method and data (.RAW and .mzML) were deposited on the MassIVE public repository and are available under the dataset accession number MSV000083475. The processing files were also added to the deposition (updates/2019-08-21_lfnothias_7cc0af40/other/1908_EMPv2_INN/). GNPS molecular networking job is available at https://gnps.ucsd.edu/ProteoSAFe/status.jsp?task=929ce9411f684cf8abd009670b293a33 and was also performed in analogue mode https://gnps.ucsd.edu/ProteoSAFe/status.jsp?task=fafdbfc058184c2b8c87968a7c56d7aa. The DEREPLICATOR jobs can be accessed at https://gnps.ucsd.edu/ProteoSAFe/status.jsp?task=ee40831bcc314bda928886964d853a52 and https://gnps.ucsd.edu/ProteoSAFe/status.jsp?task=1fafd4d4fe7e47dd9dd0b3d8bb0e6606. The SIRIUS results are available on the GitHub repository (emp/data/metabolomics/FBMN/SIRIUS). The notebooks for metabolomics data preparation and microbially related molecules establishment are available at https://github.com/lfnothias/emp_metabolomics. Amplicon and shotgun metagenomic sequence data were submitted to the European Nucleotide Archive under Project PRJEB42019 (https://www.ebi.ac.uk/ena/browser/view/PRJEB42019). Raw and demultiplexed amplicon and shotgun sequence data, the feature-table for full-length rRNA operon analysis, feature-tables for LC–MS/MS classical molecular networking and feature-based molecular networking, and the feature-table for GC–MS molecular networking data are available for download and analysis through Qiita at https://www.qiita.ucsd.edu (study: 13114). The GreenGenes database for 16S rRNA can be accessed at https://greengenes.secondgenome.com. The SILVA 138 database for 16S and 18S rRNA can be accessed at https://www.arb-silva.de. The UNITE 9 database for fungal ITS sequences can be accessed at https://unite.ut.ee. The Web of Life database can be accessed at https://biocore.github.io/wol/. The Rep200 database can be accessed at https://www.ncbi.nlm.nih.gov/refseq/. The Natural Products Atlas database can be accessed at https://www.npatlas.org. The MIBiG database can be accessed at https://mibig.secondarymetabolites.org.

## Code availability

Complete protocols for laboratory and computational workflows for both metagenomics and metabolomics data for use by the broader community are available in GitHub (https://github.com/biocore/emp/blob/master/methods/methods_release2.md).
