# Supplementary material for: Standardized multi-omics of Earth’s microbiomes reveals microbial and metabolite diversity
Source: Nat Microbiol. 2022 Nov 28;7(12):2128–50. doi: 10.1038/s41564-022-01266-x (PMC9712116; doi:10.1038/s41564-022-01266-x)
Supplement: Supplementary file 1 — Supplementary Figs 1–6 and Discussion. [file 41564_2022_1266_MOESM1_ESM.pdf]

nature microbiology

Resource

https://doi.org/10.1038/s41564-022-01266-x

# Standardized multi-omics of Earth's microbiomes reveals microbial and metabolite diversity

In the format provided by the authors and unedited

**TABLE OF CONTENTS**

1. Supplementary figures
    a. Figure S1……………………………………………………………………… 2
    b. Figure S2……………………………………………………………………… 3
    c. Figure S3……………………………………………………………………… 4
    d. Figure S4……………………………………………………………………… 5
    e. Figure S5……………………………………………………………………… 6
    f. Figure S6……………………………………………………………………… 7
2. Supplementary discussion
    a. Discussion…………………………………………………………………… 8
    b. References…………………………………………………………………… 14

## SUPPLEMENTAL FIGURES

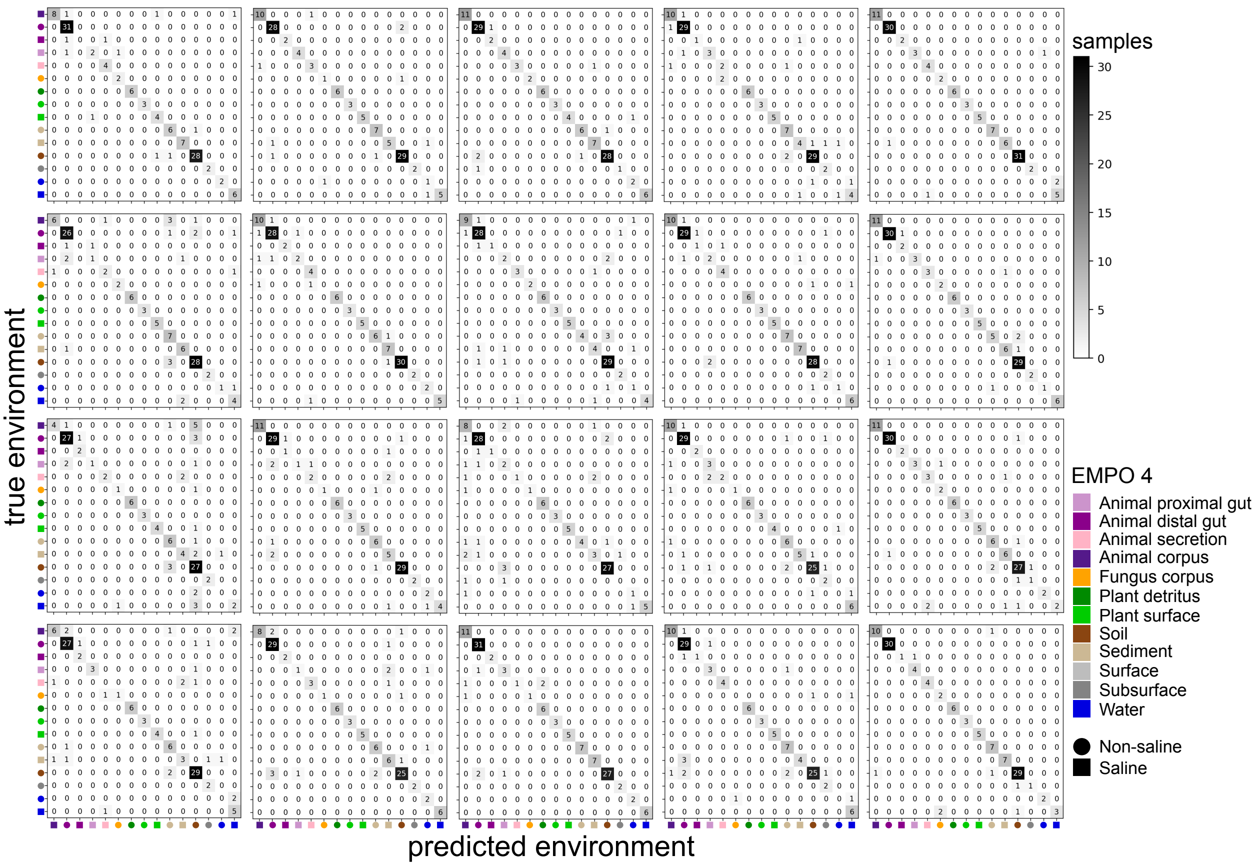


**Figure S1 | Machine learning performance for microbially-related metabolites, highlighting which environments are most often confused.** Data are from 20 iterations. The candidate confusion matrix shown in Fig. 5b of the main text is that in the first row, fifth column.

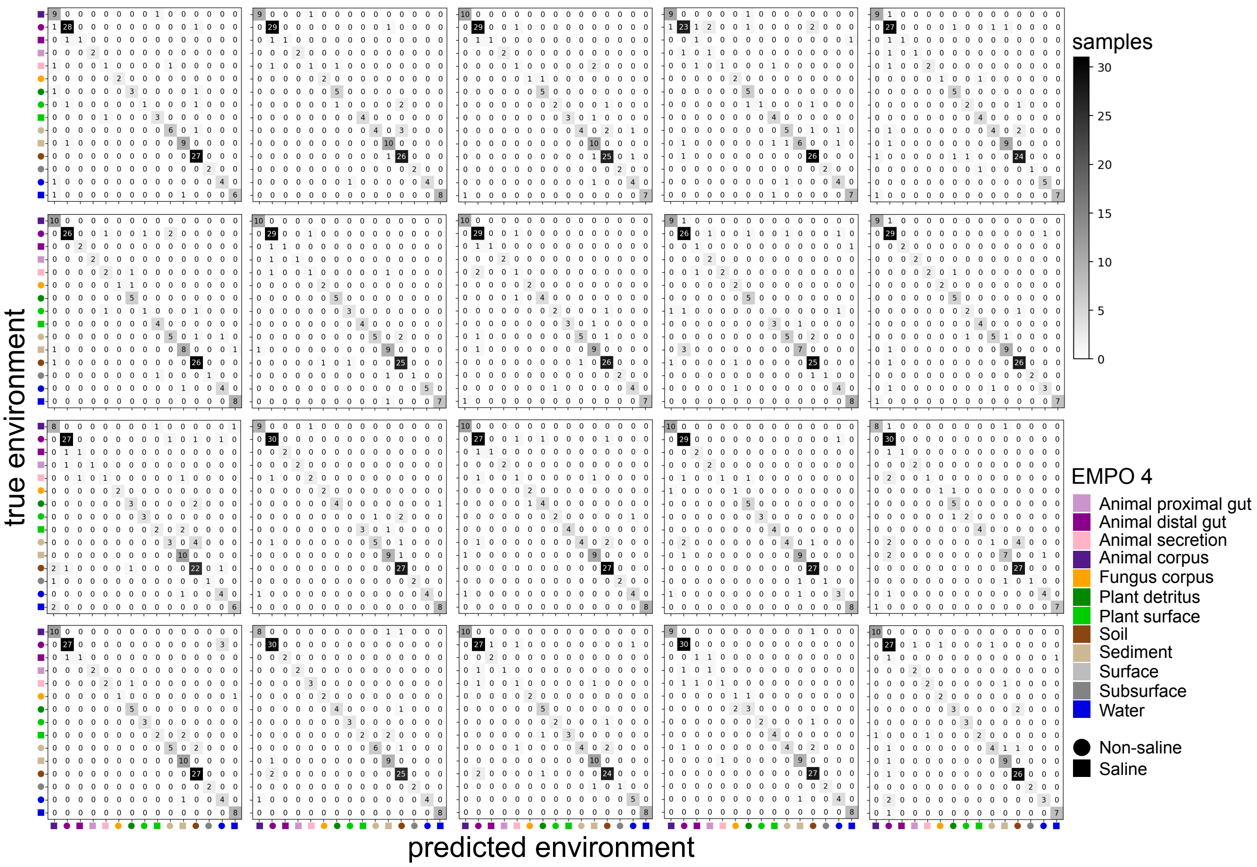


**Figure S2 | Machine learning performance for microbial taxa, highlighting which environments are most often confused.** Data are from 20 iterations. The candidate confusion matrix shown in Fig. 5b of the main text is that in the first row, third column.

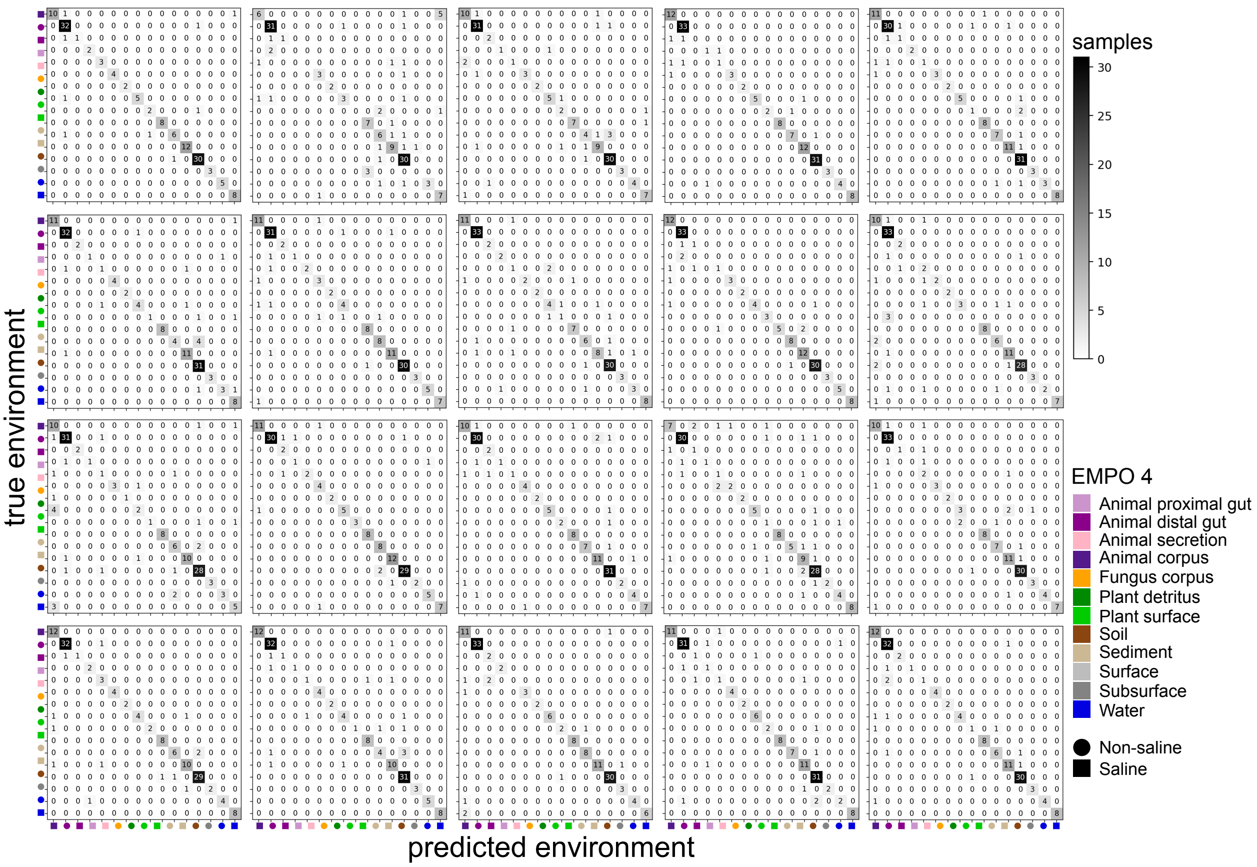


**Figure S3 | Machine learning performance for microbial functions, highlighting which environments are most often confused.** Data are from 20 iterations. The candidate confusion matrix shown in Fig. 5b of the main text is that in the first row, fourth column.

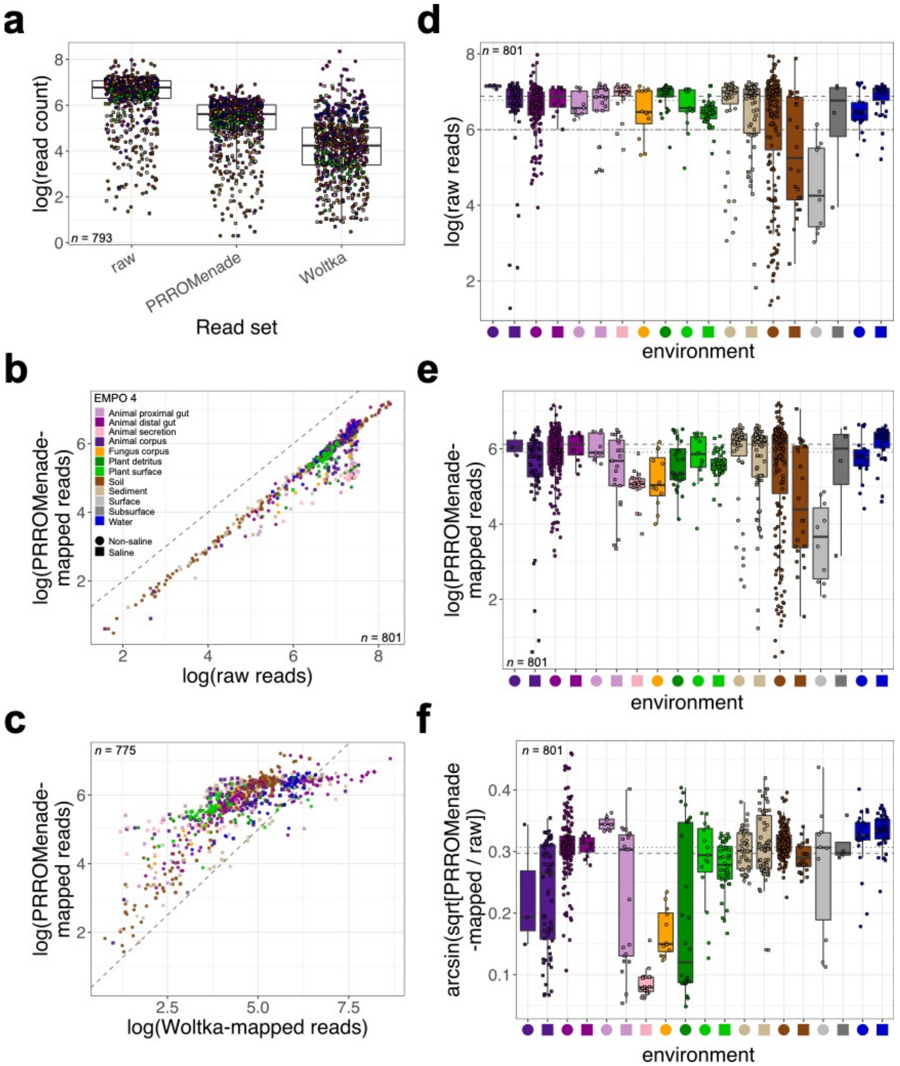


**Figure S4 | Summary of shotgun metagenomics read mapping for microbial functional profiling. a,** Comparison of read counts among raw reads, reads mapped using PRROMenade, and reads mapped using Woltka. **b,** Relationship between counts of raw reads and those mapped to using PRROMenade. The gray, dashed line indicates $y = x$. **c,** Relationship between counts of reads mapped using Woltka and those mapped using PRROMenade. The dashed line indicates $y = x$. **d**, Comparison of counts of raw reads among environments (based on EMPO 4). The two-dashed line indicates our expectation of 1 million reads from gut samples. **e,** Comparison of counts of reads mapped to using PRROMenade among environments. **f**, Comparison of the proportion of raw reads that were mapped using PRROMenade among environments. For panels **d-e**, the dashed line indicates the global mean and the dotted line indicates the global median. For all panels, note the respective *y*-axis transformation used. Colors and shapes are described in the legend in panel **b**. Boxplots are in the style of Tukey, where the center line indicates the median, lower and upper hinges the first- and third quartiles, respectively, and each whisker 1.5 x the interquartile range (IQR) from its respective hinge.

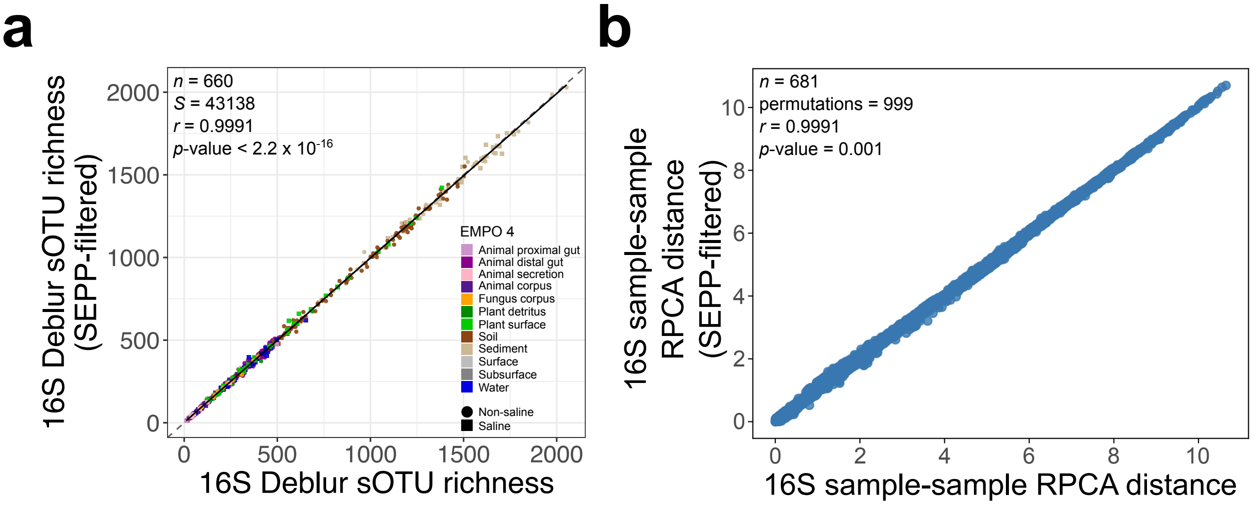


**Figure S5 | Comparison of alpha- and beta-diversity with and without removal of features that were not placed during fragment insertion (SEPP) for 16S data. a,** Spearman correlation in alpha-diversity (sOTU richness) between datasets, showing a high correlation across all environments. **b,** Mantel spearman correlation (999 permutations) in beta-diversity (sample-sample robust Aitchison distances) between datasets, showing a high correlation across all environments.

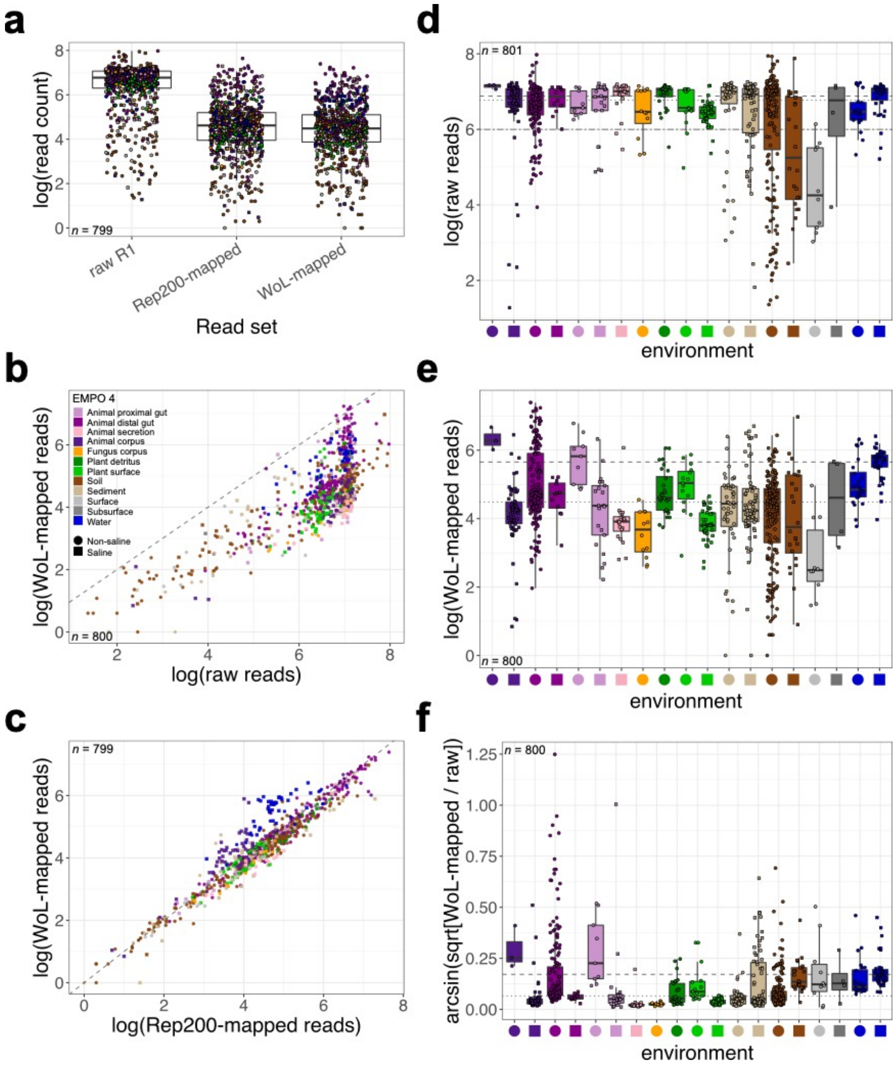


**Figure S6 | Summary of shotgun metagenomics read mapping for microbial taxonomic profiling. a,** Comparison of read counts among raw reads, reads mapped to NCBI's Rep200, and reads mapped to the Web of Life (WoL). **b,** Relationship between counts of raw reads and those mapped to the WoL. The gray, dashed line indicates $y = x$. **c,** Relationship between counts of reads mapped to NCBI's Rep200 and those mapped to the WoL. The dashed line indicates $y = x$. **d**, Comparison of counts of raw reads among environments (based on EMPO 4). The two-dashed line indicates our expectation of 1 million reads from gut samples. **e,** Comparison of counts of reads mapped to the WoL among environments. **f**, Comparison of the proportion of raw reads that were mapped to the WoL among environments. For panels **d-e**, the dashed line indicates the global mean and the dotted line indicates the global median. For all panels, note the respective *y*-axis transformation used. Colors and shapes are described in the legend in panel **b**. Boxplots are in the style of Tukey, where the center line indicates the median, lower and upper hinges the first- and third quartiles, respectively, and each whisker 1.5 x the interquartile range (IQR) from its respective hinge.

**SUPPLEMENTARY DISCUSSION**

Here, we produced as a resource a novel, multi-omics dataset comprising 880 samples that span 19 major environments, contributed by 34 principal investigators for the EMP500 (Fig. 1, Table S1). To foster additional sampling that we hope generalize our findings to additional environments and geographic locations, we expanded upon the widely-adopted set of the EMP's standardized protocols for guiding microbiome research – from sample collection to data release [1] – with new protocols for performing untargeted metabolomics and shotgun metagenomics across a diversity of sample types (Fig. 1a; Online Methods).

Across all 880 samples, we generated eight layers of data, including untargeted metabolomics and shotgun metagenomics (Table S2), providing a valuable resource for both multi-omics and meta-analyses of microbiome data. As expected, sample dropout in any one data layer was non-negligible (Table S2), reducing the number of samples in any one layer to at most roughly 500 samples (hence the EMP500). For future similar studies, we note that considering multi-omics applications during the experimental design and/or sample collection phases of studies is crucial, in part because certain metabolomics approaches are not amenable to samples stored in particular storage solutions commonly used for metagenomics (e.g., RNAlater).
We also included an example of how to apply this dataset towards addressing important questions in microbial ecological research, by describing the Earth's microbial metabolome using an integrated 'omics approach (Extended Data Fig. 1). We first explored whether every metabolite is everywhere, but the environment selects (i.e., the Baas Becking hypothesis [2,3], but for microbially-related metabolites). Our results confirm that all major groups (e.g., pathways) of metabolites are present in each environment [4], but additionally demonstrate that their relative abundances (i.e., intensity) can be limited- or enriched across environments (Fig. 2, Fig. 3a,c, Table S3). Considering the relative intensities of secondary metabolites vs. presence/absence alone drastically strengthens differences in metabolite profiles across environments for many metabolite groups including apocarotenoids, fatty acids and conjugates, glycerolipids, steroids, and polyketides (Fig. 2c,d, Fig. 3a). Interestingly, the groups of metabolites that exhibited the most obvious changes in representation were those that appeared in the fewest samples (i.e., those at low prevalence from a presence/absence perspective; e.g., carbohydrates [excluding glycosides], alkaloids) (Fig. 2a,b). Further, the environments with the most unannotated

metabolites include terrestrial animal cadavers, bioreactors that mimic the rumen of cows, freshwater, and the ocean (Fig. 2b). Similarly, environments with the most unannotated shotgun metagenomic reads for microbial functions (i.e., enzymes) included animal cadavers, marine animal secretions, terrestrial plants, and fungi (Fig. S4f). These environments merit further attention with respect to feature description, and represent valuable opportunities for the discovery of novel metabolites and functional products.

Next, we explored whether the richness and composition of metabolites in any given sample reflect those of co-occurring microbial communities. We compared alpha-diversity between metabolites and microbes, and found strong positive relationships across all samples and for many environments (Fig. 3b, Table S6). As unannotated features are included in these metrics, reference database coverage does not influence the results. Similarly, whereas estimates may be influenced by our use of rarefaction to normalize sampling effort, estimating diversity in absence of such an approach has been shown to be problematic [5]. Further, we avoided marrying estimates of alpha-diversity from our 16S vs. metagenomic data, as taxonomic profiling used a distinct reference database curated specifically for its respective data type. Future similar studies should consider the development of a reference combining both 16S and shotgun metagenomic data from bacteria and archaea. Similarly, the absence of a relationship between metabolite and microbial richness for other environments may be due to low sample representation as two lowly sampled environments, marine plant surfaces and sediments, both exhibited trends (Table S6) . We note that although input sample volumes were normalized as best as possible, the volume of sample processed may influence estimates of alpha-diversity, and that the values reported here likely exhibit some error in part due to that. In absence of technical variation, unique community carrying capacities for metabolite vs. microbes across environments may also skew trends, and we recognize that certain environments simply may not exhibit clear or underlying relationships. Still, when ranking environments based on alpha-diversity, certain patterns are clear. For example, in addition to confirming previous observations that marine sediments are one of the most microbially diverse environments on Earth [6], we showed that marine sediments are also the most metabolically diverse (Fig. 3b). To our knowledge, this is the first assessment of the metabolic alpha-diversity in marine sediments as it relates to microbial diversity in those samples in a context including other diverse environments. Although not the most lacking with respect to

annotation rates for metabolites, the high diversity in marine sediments merits further exploration of those molecules.

We also found a strong correlation in sample–sample distances between metabolite and microbial datasets (Table 1), and significant turnover of features across environments (Fig. 3c,d). For both metabolites and microbial taxa, the effect of host-association was much stronger than salinity in explaining variation in community composition (Fig. 3c,d). We also observed a much weaker influence of salinity in separating samples based on metabolites vs. microbes (Fig. 3c,d). Together, these findings support recognizing host-association as EMPO 1, and confirm our prediction that environmental similarity between datasets may be distinct when based on one dataset vs. the other. This may indicate that although microbial cells respond strongly to salinity gradients, the taxa they represent can have similar metabolic profiles. Our hypothesis is supported by our observation that co-occurrences with metabolites appear to be more structured by environment than phylogeny for microbial taxa (Extended Data Fig. 9). Additional support lies in our finding that the differential intensities of important pathways and superclasses are highly similar between freshwater and marine environments (Fig. 3a), and that the same groups of metabolites can occur in both habitats yet are associated with distinct microbes within each (Fig. 5e,f).

The lack of complete turnover in metabolites vs. microbes with respect to the environment generated unique patterns of nestedness between datasets (Extended Data Fig. 5, Extended Data Fig. 6). Whereas nestedness patterns among environments with respect to microbial taxon profiles matched our expectations based on assembly dynamics and dispersal patterns (e.g., host-associated communities are a subset of free-living ones), as well as previous observations based on 16S data [6], those based on metabolite profiles were more weakly correlated with our description of environments based on EMPO. This may be in part due to the weaker effect of salinity on sample beta-diversity for metabolites (Extended Data Fig. 5a, Extended Data Fig. 6a), and similarity in metabolite profiles among microbes from disparate environments (Fig. 5d-f). It may also indicate that microbially-related metabolites assembly and structure uniquely from the microbes that produce them in nature. Nevertheless, future efforts to expand database coverage for metabolites should consider this, as the expectation that diversity will continue to increase with sampling of these distinct environments may not be realized.

Given that profiles for metabolites and microbes were habitat-specific, we used machine-learning to identify several metabolites, microbial taxa, and microbial functions that could accurately classify samples among environments (Fig. 4, Extended Data Fig. 7a, Table S7). Overall accuracy for each dataset was ≥88% (Fig. 4, Extended Data Fig. 7a), confirming our prediction that certain features could distinguish among environments. Although infrequent among 20 iterations, certain environments were occasionally confused (Extended Data Fig. 7b, Figs. S1-S3). For example, when based on metabolites, marine animal proximal gut was once misclassified as seawater, marine sediment was once misclassified as non-saline animal distal gut, freshwater was twice misclassified as seawater, and seawater was once misclassified as marine animal secretion (i.e., during a single iteration) (Extended Data Fig. 7b). We note that the majority of misclassifications were between compositionally similar environments (Extended Data Fig. 7b, Figs. S1-S3). Features identified here as important for classification should prove useful as indicators of particular environments (Fig. 4, Table S7), which can be used for applications such as source tracking [7] and forensics [8]. When considering the twenty most highly ranked metabolites regarding impacting classification performance, metabolites classified to the pathways amino acids and peptides were not present (Fig. 4a), although metabolites from this pathway were differentially abundant across samples (Fig. 3c, Table S3, Table S4). Rather, the most abundant and highly ranked pathway among those highly predictive metabolites was for terpenoids, highlighting the importance of this group of metabolites in distinguishing Earth's environments (Fig. 4a, Table S7). Terpenoids are the largest class of natural products recognized to date, and are known to be the most prevalent secondary metabolites in nature [9], which we also showed with our presence/absence data (Fig. 2a). Although known most commonly from plants, recent work has described a diversity of terpenoids produced by microbes, which range in activity from stress responses to signaling and communication [9]. Future work should aim to further characterize the terpenoids discovered in this dataset.

We also identified metabolite–microbe co-occurrences, and as a first step towards characterizing them as salient features of the environment, showed that these relationships can be specific to certain habitats (Fig. 5, Extended Data Fig. 4). We view strongly co-occurring metabolite–microbe pairs as features of the environment that in part can be grappled for further exploration, for example as predictors in models of environmental change [10]. We demonstrated

that both distinct metabolite pathways (e.g., carbohydrates vs. terpenoids) and metabolites within the same pathway (e.g., two groups of fatty acids), can be used to distinguish environments based on their co-occurrences with microbes (Fig. 6c-f, Extended Data Fig. 4f-j). Similarly, we showed that certain metabolites and microbes have an especially high number of strong co-occurrences with one another (Extended Data Fig. 8, Extended Data Fig. 9). We hypothesize that microbes co-occurring with relatively many metabolites represent 'chemically-talented' taxa that may be useful for discovery of novel compounds. Further culture-based studies should continue to explore and characterize the metabolic diversity among these microbes.

Our results highlight the advantages of using standardized methods to interpret and predict the contributions of microbes and their environments to chemical profiles in nature. Standardization of methods is of utmost importance, as no single lab can sample everything, and because a multitude of methods for performing a microbiome study exist [11-13]. Due to inherent biases among distinct methods such as towards describing particular taxa [13,14], such lack of standardization prevents robust meta-analysis [6,15,16]. Issues surrounding such bias extend from sequencing to metabolomics, which may be subject to greater technical variation due in part to unavoidable batch effects and use of extraction methods unique to particular sample types [17]. The EMP500 overcomes these challenges by using standardized approaches, allowing for robust tracking of microbes and metabolites that permits the description of features that distinguish one habitat from another. This insight fosters understanding of the processes that make each habitat unique, and that may be vital to the functional diversity in the environment. By using standardized methods for sample processing and data analysis, the EMP500 allows for additional contributions, further expanding our insight into these communities.

We argue that using only sequence-based approaches to interpret functional potential can be misleading, as the presence of genomic loci and/or transcripts does not equate to the presence of a functional product in the environment. Using our presence/absence data for metabolites, we observed a trend in the uniform distribution of metabolite pathways across environments (Fig. 2a,c). However, when taking into account the relative intensities of metabolites – to date only possible using metabolomics – we observed significant differences in the distribution of particular groups of metabolites across the Earth's environments (Fig. 2b,d, Fig. 3a). This emphasizes the utility and importance of directly measuring functional products in the

environment, rather than estimating their potential from underlying genomic elements. We note that the uniform distributions of metabolite pathways and superclasses across environments based on presence/absence data (Fig. 2a,c) are similar to previous observations based on BGC annotation of a global dataset of MAGs [4]. It could be that abundance/intensity data for the products of BGCs may provide a different view, as they have here. We also recognize that using only metabolomics-based approaches can make the detection of certain molecules difficult, as some metabolites have relatively short lifespans, are consumed rapidly, and/or are cycled between members of the community therefore escaping detection [18,19].

Beyond the important ecological questions explored here, several others such as those surrounding host-microbe interactions, microbial ecology in a changing world, and environmental processes merit future exploration [20]. In some cases, addressing these questions will only be feasible following the collection of additional samples that span additional environments and/or geographic locations. For example, although we explored turnover and nestedness, one major question is whether these communities conform to the same biogeographic and ecological principles as in other types of communities, such as those of animals or plants [20-22]. For example, we were unable to explore whether our features follow the latitudinal diversity gradient. The increase in species richness towards lower latitudes is apparent in many populations including those of several animals and plant species, but also planktonic marine bacteria [23] and soilborne *Streptomyces* [24]. This trend has been less-explored at the community level, outside of soils [25,26]. Although highly host-specific groups such as ectomycorrhizal fungi do not follow this gradient due to the distributions of their host populations [27], it is unclear what pattern metabolites and microbes exhibit, and whether there is variation among all of the environments recognized here. As another example, we did not explicitly explore the importance of rare features with respect to differences among environments. In addition to rare features serving as potential indicators of particular interactions [28] or ecological trends [29,30], little is known regarding the relationships between rare features from distinct data layers (e.g., metabolites and microbial taxa). Although we might expect metabolites produced by rare microbes to also be rare in the environment, the suite of community interactions acting on those metabolites may alter distributions in context-dependent ways.

**References.**

1. Thompson, L. *et al.* EMP Sample Submission Guide v1. *protocols.io* (2018) doi:10.17504/protocols.io.pfqdjmw.
2. Baas Becking, L. G. M. Geobiologie of inleiding tot de milieukunde. The Hague, the Netherlands: W. P. Van Stockum & Zoon (in Dutch) (1934).
3. de Wit, R. and Bouvier, T. 'Everything is everywhere but the environment selects'; what did Baas Becking and Beijerinck really say? *Environ. Microbiol.* **8**, 755-758 (2006). doi: 10.1111/j.1462-2920.2006.01017.x
4. Nayfach, S. *et al.* A genomic catalog of Earth's microbiomes. *Nat. Biotechnol.* (2020) doi:10.1038/s41587-020-0718-6
5. Weiss, S. *et al.* Normalization and microbial differential abundance strategies depend on data characteristics. *Microbiome* **5**, 27 (2017). doi: 10.1186/s40168-017-0237-y
6. Thompson, L. R. *et al.* A communal catalogue reveals Earth's multiscale microbial diversity. *Nature* **551**, 457–463 (2017). doi: 10.1038/nature24621
7. Knights, D. *et al.* Bayesian community-wide culture-independent microbial source tracking. *Nat. Methods* **8**, 761-763 (2011). doi: 10.1038/nmeth.1650
8. Lax, S. *et al.* Forensic analysis of the microbiome of phones and shoes. *Microbiome* **3**, 21 (2015). doi: 10.1186/s40168-015-0082-9
9. Avalos, M. *et al.* Biosynthesis, evolution and ecology of microbial terpenoids. *Nat. Prod. Rep.* **39**, 249 (2022). doi: 10.1039/d1np00047k
10. Reid, A. Incorporating microbial processes into climate models: Report on an American Academy of Microbiology Colloquium held on Feb. 21-23, 2011. Washington (DC): American Society for Microbiology; (2011). doi: 10.1128/AAMCol.21Feb.2011
11. Di Bella, J. M. *et al.* High throughput sequencing methods and analysis for microbiome research. *J. Microbiol. Meth.* **95**, 401-414 (2013). doi: 10.1016/j.mimet.2013.08.011
12. Byrd, D. A. *et al.* Comparison of methods to collect fecal samples for microbiome studies using whole-genome shotgun metagenomic sequencing. *mSphere* **5**, e00827-19 (2020). doi: 10.1128/mSphere.00827-19

13. Shaffer, J. P. *et al.* A comparison of six DNA extraction protocols for 16S, ITS, and shotgun metagenomic sequencing of microbial communities. *BioTechniques* **73**, 2022-0032 (2022) doi: 10.2144/btn-2022-0032
14. McLaren, M. R. Consistent and correctable bias in metagenomic sequencing experiments. *eLife* **8**, e46923 (2019). doi: 10.7554/eLife.46923
15. Knight, R. *et al.* Best practices for analysing microbiomes. *Nat. Rev. Microbiol.* **16**, 410-422 (2018). doi: 10.1038/s41579-018-0029-9
16. Vangay, P. *et al.* Microbiome metadata standards: report of the national microbiome data collaborative's workshop and follow-on activities. *mSystems* **6**, e01194-20 (2021). doi: 10.1128/mSystems.01194-20
17. De Livera, A. M. *et al.* Statistical methods for handling unwanted variation in metabolomics data. *Anal. Chem.* **87**, 3606-3615 (2015). doi: 10.1021/ac502439y
18. Lu, W. *et al.* Metabolite measurement: pitfalls to avoid and practices to follow. *Annu. Rev. Biochem.* **86**, 277-304 (2017). doi: 10.1146/annurev-biochem-061516-044952
19. Pinu, F. R. *et al.* Analysis of intracellular metabolites from microorganisms: quenching and extraction protocols. *Metabolites* **7**, 53 (2017). doi: 10.3390/metabo7040053
20. Antwis, R. E. Fifty important research questions in microbial ecology. *FEMS Microbiol. Ecol.* **93**, fix044 (2017). doi: 10.1093/femsec/fix044
21. Prosser, J. I. *et al.* The role of ecological theory in microbial ecology. *Nat. Rev. Microbiol.* **5**, 384-392 (2007). doi: 10.1038/nrmicro1643
22. Dickey, J. R. *et al.* The utility of macroecological rules for microbial biogeography. *Front. Ecol. Evol.* **9**, 633155 (2021). doi: 10.3389/fevo.2021.633155
23. Fuhrman, J. A. *et al.* A latitudinal diversity gradient in planktonic marine bacteria. *PNAS* **105**, 7774-7778 (2008). doi: 10.1073/pnas.0803070105
24. Andam, C. P. *et al.* A latitudinal diversity gradient in terrestrial bacteria in the genus *Streptomyces. mBio* **7**, e02200 (2016). doi: 10.1128/mBio.02200-15
25. Zhang, X. *et al.* Local community assembly mechanisms shape soil bacterial β diversity patterns along a latitudinal gradient. *Nat. Comm.* **11**, 5428 (2020). doi: 10.1038/s41467-020-19228-4

26. Xiao, X. *et al.* A latitudinal gradient of microbial β-diversity in continental paddy soils. *Global Ecol. Biogeog.* **30**, 909-919 (2021). doi: 10.1111/geb.13267
27. Tedersoo, L. and Nara, K. Latitudinal gradient of biodiversity is reversed in ectomycorrhizal fungi. *New Phytol.* **185**, 351-354 (2010). doi:
28. Ainsworth, T. D. *et al.* The coral core microbiome identified rare bacterial taxa as ubiquitous endosymbionts. *ISME J.* **9**, 2261-2274 (2015). doi: 10.1038/ismej.2015.39
29. Oono, R. *et al.* Distance decay relationships in foliar fungal endophytes are driven by rare taxa. *Environ. Microbiol.* **19**, 2794-2805 (2017). doi: 10.1111/1462-2920.13799
30. Reveillaud, J. *et al.* Host-specificity among abundant and rare taxa in the sponge microbiome. *ISME J.* **8**, 1198-1209 (2014). doi: 10.1038/ismej.2013.227
