# Supplementary material for: Standardized multi-omics of Earth’s microbiomes reveals microbial and metabolite diversity
Source: Nat Microbiol. 2022 Nov 28;7(12):2128–50. doi: 10.1038/s41564-022-01266-x (PMC9712116; doi:10.1038/s41564-022-01266-x)
Supplement: Supplementary file 2 — Reporting Summary [file 41564_2022_1266_MOESM2_ESM.pdf]

nature portfolio

Corresponding author(s): Rob Knight (robknight@ucsd.edu)

Last updated by author(s): Sep 23, 2022

Reporting Summary

Nature Portfolio wishes to improve the reproducibility of the work that we publish. This form provides structure for consistency and transparency in reporting. For further information on Nature Portfolio policies, see our Editorial Policies and the Editorial Policy Checklist.

Statistics

For all statistical analyses, confirm that the following items are present in the figure legend, table legend, main text, or Methods section.

n/a	Confirmed	
☐	☒	The exact sample size (*n*) for each experimental group/condition, given as a discrete number and unit of measurement
☐	☒	A statement on whether measurements were taken from distinct samples or whether the same sample was measured repeatedly
☐	☒	The statistical test(s) used AND whether they are one- or two-sided<br>*Only common tests should be described solely by name; describe more complex techniques in the Methods section.*
☐	☒	A description of all covariates tested
☐	☒	A description of any assumptions or corrections, such as tests of normality and adjustment for multiple comparisons
☐	☒	A full description of the statistical parameters including central tendency (e.g. means) or other basic estimates (e.g. regression coefficient) AND variation (e.g. standard deviation) or associated estimates of uncertainty (e.g. confidence intervals)
☐	☒	For null hypothesis testing, the test statistic (e.g. *F*, *t*, *r*) with confidence intervals, effect sizes, degrees of freedom and *P* value noted<br>*Give P values as exact values whenever suitable.*
☒	☐	For Bayesian analysis, information on the choice of priors and Markov chain Monte Carlo settings
☐	☒	For hierarchical and complex designs, identification of the appropriate level for tests and full reporting of outcomes
☐	☒	Estimates of effect sizes (e.g. Cohen's *d*, Pearson's *r*), indicating how they were calculated

Our web collection on statistics for biologists contains articles on many of the points above.

Software and code

Policy information about availability of computer code

Data collection	We provide complete protocols for laboratory- and computational workflows for both metagenomics and metabolomics data collection for use by the broader community, available on GitHub (https://github.com/biocore/emp/blob/master/methods/methods_release2.md).
Data analysis	We provide complete protocols for laboratory- and computational workflows for both metagenomics and metabolomics data analysis for use by the broader community, available on GitHub (https://github.com/biocore/emp/blob/master/methods/methods_release2.md). Software for data analysis included: ZebraDesigner Pro 3; ProteoWizard v3.0.19; MZmine 2; SIRIUS v4.4.25 (includes ZODIAC, CANOPUS, CSI:FingerID; DEREPLICATOR+; QIIME2-2020.6; R v4.0.0; bowtie2 v2.3.2; Woltka v0.1.4; songbird v1.0.4; and mmvec v1.0.6.

For manuscripts utilizing custom algorithms or software that are central to the research but not yet described in published literature, software must be made available to editors and reviewers. We strongly encourage code deposition in a community repository (e.g. GitHub). See the Nature Portfolio guidelines for submitting code & software for further information.

Data

Policy information about availability of data

All manuscripts must include a data availability statement. This statement should provide the following information, where applicable:

- Accession codes, unique identifiers, or web links for publicly available datasets
- A description of any restrictions on data availability
- For clinical datasets or third party data, please ensure that the statement adheres to our policy

The mass spectrometry method and data (.RAW and .mzML) were deposited on the MassIVE public repository and are available under the dataset accession number MSV000083475. The processing files were also added to the deposition (updates/2019-08-21_lfnothias_7cc0af40/other/1908_EMPv2_INN/). GNPS molecular networking job is available at https://gnps.ucsd.edu/ProteoSAFe/status.jsp?task=929ce9411f684cf8abd009670b293a33 and was also performed in

analogue mode https://gnps.ucsd.edu/ProteoSAFe/status.jsp?task=fafdbfc058184c2b8c87968a7c56d7aa. The DEREPLICATOR jobs can be accessed here: https://gnps.ucsd.edu/ProteoSAFe/status.jsp?task=ee40831bcc314bda928886964d853a52 and https://gnps.ucsd.edu/ProteoSAFe/status.jsp?task=1fafd4d4fe7e47dd9dd0b3d8bb0e6606. The SIRIUS results are available on the GitHub repository (https://github.com/biocore/emp/tree/master/data/metabolomics/FBMN/SIRIUS). The notebooks for metabolomics data preparation and microbially-related molecules establishment are available on this repository (https://github.com/lfnothias/emp_metabolomics). Amplicon and shotgun metagenomic sequence data are submitted to the European Nucleotide Archive under Project: PRJEB42019 (https://www.ebi.ac.uk/ena/browser/view/PRJEB42019). Raw and demultiplexed amplicon and shotgun sequence data, the feature-table for full-length rRNA operon analysis, feature-tables for LC-MS/MS classical molecular networking and feature-based molecular networking, and the feature-table for GC-MS molecular networking data are available for download and analysis through Qiita at https://www.qiita.ucsd.edu (study: 13114). The GreenGenes database for 16S rRNA can be accessed at https://greengenes.secondgenome.com. The SILVA 132 database for 16S and 18S rRNA can be accessed at https://www.arb-silva.de. The UNITE 8 database for fungal ITS sequences can be accessed at https://unite.ut.ee. The Web of Life database of microbial genomes can be accessed at https://biocore.github.io/wol/. The Rep200 database can be accessed at https://www.ncbi.nlm.nih.gov/refseq/. The Natural Products Atlas database can be accessed at https://www.npatlas.org. The MIBiG database can be accessed at https://mibig.secondarymetabolites.org.

Field-specific reporting

Please select the one below that is the best fit for your research. If you are not sure, read the appropriate sections before making your selection.

☐ Life sciences ☐ Behavioural & social sciences ☒ Ecological, evolutionary & environmental sciences

For a reference copy of the document with all sections, see nature.com/documents/nr-reporting-summary-flat.pdf

Ecological, evolutionary & environmental sciences study design

All studies must disclose on these points even when the disclosure is negative.

Study description	This study is a multi-omic survey of a diverse range of microbial environments on planet Earth, spanning host-associated and free-living environments according to a pre-determined sample type ontology. A total of n=880 samples were processed as described next.
Research sample	Samples were chosen to span a wide range of microbial environments. The number (n) of samples in each EMPO level 3 (version 1) category were as follows: Soil (non-saline) 242, Animal distal gut 184, Plant surface 87, Animal corpus 67, Sediment (saline) 66, Sediment (non-saline) 47, Water (saline) 39, Water (non-saline) 30, Animal proximal gut 30, Plant corpus 28, Subsurface (non-saline) 24, Animal secretion 20, Fungus corpus 12, Surface (saline) 2, Surface (non-saline) 2.
Sampling strategy	A call was placed to microbiome researchers around the world to propose and submit microbiome samples for a global survey. Effort was made to span a diverse range of environments, and the EMP Ontology (EMPO) was created to capture relevant axes of microbial environment diversity. All environments were represented but not necessarily with the same number of samples. In cases where even sampling was required for statistical analysis, subsampling or normalization was applied.
Data collection	Data were acquired using standard metagenomics and metabolomics procedures (see methods) by Jon Sanders and Greg Humphrey (amplicon- and shotgun metagenomic sequences), Louis-Felix Nothias (LC-MS/MS), and Sneha Couvillion (GC-MS).
Timing and spatial scale	Data collection for each method was done across all samples simultaneously in order to reduce or eliminate batch effects.
Data exclusions	In cases where even sampling was required for statistical analysis, subsampling or normalization was applied. In these cases certain samples and/or microbial or metabolite features were excluded at random.
Reproducibility	Samples were randomly allocated to plates for each analysis method to avoid batch effects. Multiple sequencing runs were incorporated to confirm patterns in metagenomic data. Additionally, most of the sample types and studies provided replicate samples for treatments.
Randomization	In cases where even sampling was required for statistical analysis, samples were randomly subsampled or normalized.
Blinding	Samples were given non-descriptive sample names for data collection and data analysis. Sample groups were only identified at the final data visualization step.

Did the study involve field work? ☐ Yes ☒ No

Reporting for specific materials, systems and methods

We require information from authors about some types of materials, experimental systems and methods used in many studies. Here, indicate whether each material, system or method listed is relevant to your study. If you are not sure if a list item applies to your research, read the appropriate section before selecting a response.

Materials & experimental systems

n/a	Involved in the study
☒	☐ Antibodies
☒	☐ Eukaryotic cell lines
☒	☐ Palaeontology and archaeology
☒	☐ Animals and other organisms
☒	☐ Human research participants
☒	☐ Clinical data
☒	☐ Dual use research of concern

Methods

n/a	Involved in the study
☒	☐ ChIP-seq
☒	☐ Flow cytometry
☒	☐ MRI-based neuroimaging
